# Global Mass Spectrometry Based Metabolomics Profiling of Erythrocytes Infected with *Plasmodium falciparum*

**DOI:** 10.1371/journal.pone.0060840

**Published:** 2013-04-09

**Authors:** Theodore R. Sana, D. Benjamin Gordon, Steven M. Fischer, Shane E. Tichy, Norton Kitagawa, Cindy Lai, William L. Gosnell, Sandra P. Chang

**Affiliations:** 1 Life Sciences Group, Agilent Technologies, Santa Clara, California, United States of America; 2 Department of Tropical Medicine, Medical Microbiology and Pharmacology, John A. Burns School of Medicine, University of Hawaii at Manoa, Honolulu, Hawaii, United States of America; University of South Alabama, United States of America

## Abstract

Malaria is a global infectious disease that threatens the lives of millions of people. Transcriptomics, proteomics and functional genomics studies, as well as sequencing of the *Plasmodium falciparum* and *Homo sapiens* genomes, have shed new light on this host-parasite relationship. Recent advances in accurate mass measurement mass spectrometry, sophisticated data analysis software, and availability of biological pathway databases, have converged to facilitate our global, untargeted biochemical profiling study of *in vitro P. falciparum*-infected (IRBC) and uninfected (NRBC) erythrocytes. In order to expand the number of detectable metabolites, several key analytical steps in our workflows were optimized. Untargeted and targeted data mining resulted in detection of over one thousand features or chemical entities. Untargeted features were annotated via matching to the METLIN metabolite database. For targeted data mining, we queried the data using a compound database derived from a metabolic reconstruction of the *P. falciparum* genome. In total, over one hundred and fifty differential annotated metabolites were observed. To corroborate the representation of known biochemical pathways from our data, an inferential pathway analysis strategy was used to map annotated metabolites onto the BioCyc pathway collection. This hypothesis-generating approach resulted in over-representation of many metabolites onto several IRBC pathways, most prominently glycolysis. In addition, components of the “branched” TCA cycle, partial urea cycle, and nucleotide, amino acid, chorismate, sphingolipid and fatty acid metabolism were found to be altered in IRBCs. Interestingly, we detected and confirmed elevated levels for cyclic ADP ribose and phosphoribosyl AMP in IRBCs, a novel observation. These metabolites may play a role in regulating the release of intracellular Ca^2+^ during *P. falciparum* infection. Our results support a strategy of global metabolite profiling by untargeted data acquisition. Untargeted and targeted data mining workflows, when used together to perform pathway-inferred metabolomics, have the benefit of obviating MS/MS confirmation for every detected compound.

## Introduction

The past decade has seen an increase in international funding for malaria control as it remains one of the world’s most significant infectious diseases, with approximately 225 million malaria cases and 781,000 malaria deaths reported in 2009 [1]. In light of recent news that resistance to the highly-effective drug, artemisinin appears to be spreading in Southeast Asia [2], while the prevalence of drug resistance to other anti-malarial drugs is increasing [3], strategies to identify anti-malarial compounds with novel mechanisms of action are needed.

Metabolomics is the profiling of the total set of metabolites, or low molecular weight biochemical intermediates, resulting from the physiological, developmental or pathological state of a cell, tissue, organ or organism [4]–[6]. Metabolomics has emerged as an important field of research, hastening the development of new accurate mass databases, MS/MS spectral libraries and complex bioinformatics software [7]. The confluence of several key enabling technologies: liquid and gas chromatography, mass spectrometry, genome sequencing, nuclear magnetic resonance spectroscopy, and the continuously evolving speed and storage capacity of computers, have resulted in dramatic progress in this field. As a result, untargeted, global profiling of malaria infected RBCs has the potential to become a viable analytical approach for malaria drug development.

Biochemical studies of *Plasmodium falciparum*, the most lethal of the four parasite species in humans, have been complemented by more recent bioinformatics analyses of the malaria parasite genome, transcriptome, and proteome [8]–[11]. This information has been compiled to construct an organism-specific metabolic pathway database (Malaria Parasite Metabolic Pathways, MPMP) [12]. Based in part on the general metabolic pathways described in the Kyoto Encyclopedia of Genes and Genomes (KEGG) [13], MPMP integrates features specific to *Plasmodium* metabolism. The complete sequencing of both the human [14] and *P. falciparum* genomes [8] has stimulated a wide variety of functional genomics research, such that it is now possible to gain further insight into mechanisms of host:parasite interactions. Nevertheless, approximately 55% of the protein-coding genes within the *P. falciparum* genome are of unknown function (PlasmoDB 7.2 ApiCyc database [15]), and are likely to encode components of new metabolic pathways.

Host-parasite interactions in malaria are complicated by life cycles in vertebrate and invertebrate hosts, as well as distinct stages associated with different tissues within each of these hosts. This manuscript describes a strategy for global metabolomic profiling of the intracellular protozoan, *P. falciparum*, and presents extensive data on the metabolome of *P. falciparum*-infected erythrocytes. Differences in global metabolite abundance profiles between normal and *P. falciparum*-infected erythrocytes were evaluated using an untargeted data acquisition approach. Since no single method currently exists that is sufficiently comprehensive to extract all metabolites, we developed a robust biphasic liquid-liquid metabolite extraction and sample processing protocol that included three different pH-adjusted solvents to facilitate extraction of metabolites with pH-dependent solubilities [16]. Hydrophobic and amphipathic metabolites were readily separated by Reverse-Phase (RP) chromatography, while hydrophilic metabolites were recovered by Aqueous Normal Phase (ANP) chromatography [17], [18]. We complemented LC analyses with GC/MS, primarily for sugars and volatile compounds, via retention time locked (RTL) matching to a spectral library of standards [19].

Two distinct bioinformatics approaches were employed for finding compounds in LC-MS data: (1) a global, untargeted discovery-driven workflow using an unbiased feature finding algorithm for extracting hundreds of chromatographic peaks, annotating peaks via database matching and mapping annotated entities onto biological pathways to find biochemical signatures separating NRBCs from IRBCs [20], and (2) a targeted data mining approach employing an algorithm that searches for features using a list of empirical formulas downloaded from a *P. falciparum* database of metabolites. Pathway visualization software was used for applying an inferred pathway mining interpretation of annotated metabolites. This aided in determining the relative representation of pathway-associated metabolites after querying and mapping them onto *P. falciparum* 3D7 pathways downloaded from the SRI registry of Pathway/Genome Databases (http://biocyc.org/registry.html). In this way, no *a priori* assumptions were made regarding the outcome of untargeted experiments. Inferential pathway associations based on annotated metabolite enrichment were then used to help guide the selection of specific metabolites for subsequent MS/MS identification and/or RT matching of metabolites to a library of chemical standards.

Untargeted data mining resulted in over a thousand independent features per sample. These features were then annotated via METLIN database matching. Targeted data mining resulted in a more limited list of features, but included some features that were not detected using the untargeted approach. Our results demonstrate high value in employing a broad metabolite extraction strategy, coupled with different chromatographic separation technologies, and MS-based multi-mode detection. Moreover, combining a dual data mining strategy with an inferred pathway enrichment approach reduces the number of compounds requiring identification and validation and will inform the selection of pathway proteins for subsequent targeted peptide multiple reaction monitoring (MRM) validation.

## Materials and Methods

*Refer to Methods S1 for additional details.*

### Ethics Statement

Collection of human blood samples for this study was conducted according to the principles expressed in the Declaration of Helsinki and under protocols approved by the Human Use Research Committee of the University of Hawaii. All subjects provided written informed consent for the collection of samples and subsequent analysis.

### *P. falciparum* in vitro Cultivation

The 3D7 strain of *Plasmodium falciparum* was grown in human erythrocytes in RPMI 1640 medium supplemented with 25 mM HEPES, 20 mM sodium bicarbonate, 370 µM hypoxanthine and 0.5% AlbuMAX (Invitrogen) [21], [22]. Since gene expression throughout the intraerythrocytic cycle of *P. falciparum* is thought to be variable and highly regulated [10], [23], an asynchronous culture was analyzed to maximize the range of features detected. Asynchronous cultures of *P. falciparum* infected red blood cells (IRBC) and control non-infected red blood cells (NRBC) were established at an initial hematocrit of 5% in 12-well cell culture plates at 37°C under an atmosphere of 90% N_2_, 5% O_2_, and 5% CO_2_. Cultures were maintained in this manner with daily media changes until the IRBC parasitemia reached approximately 10%. The NRBC and the IRBC cultures were incubated in the absence or presence of 250 units streptolysin O or SLO (Sigma-Aldrich, St. Louis, MO) per ml of culture for 30 minutes at 37°C to selectively permeabilize non-infected cells [24] and consequently enrich for metabolites in IRBCs. The concentration of SLO selected for these experiments was observed to alter membrane permeability but not result in RBC lysis, with the intention of allowing diffusion of metabolites out of NRBC without affecting the overall biomass. Altered permeability was monitored microscopically by evaluating the conversion of erythrocytes from a biconcave to a spherical morphology.

### Study Design

Four separate biological replicate cultures containing 0.5 mL of cells were prepared for each of the three pH adjusted solvent extracts, totaling 48 samples equally divided between the conditions: (1) NRBC, SLO untreated, (2) NRBC, SLO treated, (3) IRBC, SLO untreated, and (4) IRBC, SLO treated. A separate aliquot was separated by Aqueous Normal Phase (ANP) chromatography and analyzed by LC-ESI - and LC-ESI +. All 48 samples were analyzed by GC/MS.

### Sample Processing

Samples were processed according to a biphasic liquid-liquid extraction (LLE) protocol [16]. Briefly, RBC metabolism was quenched by extracting metabolites into methanol/water/chloroform (4∶1∶3) solvents adjusted to pH 2, 7 or 9. To remove residual proteins that would otherwise affect retention time reproducibility and lead to detection interference in LC/MS, aqueous phase proteins were precipitated with an equal volume of acetonitrile, and depleted by centrifugation and filtration. The aqueous phase extracts were filtered through pre-washed 0.2 µm Pall Nanosep MF filters (Pall Life Sciences, Ann Arbor, MI), followed by 10 kDa and then 3 kDa Pall Nanosep centrifugal device filters, briefly dried under vacuum, and re-suspended in LC solvent: (1) for RP separation, pellet was resuspended in 100 µL of 50∶50 methanol:water, 0.2% acetic acid, and (2) for ANP separation, pellet was resuspended in 100 µL of 70∶30 acetonitrile:water, 0.1% formic acid.

### Analytical Workflow

Briefly, (1) metabolite recovery was maximized by using different organic extraction solvents that were adjusted to pH 2, 7, and 9; (2) protein precipitation was followed by filtration of metabolite extracts in order to minimize protein carryover; (3) addition of unlabeled inorganic external standards: 9-Anthracene Carboxylic acid and 1-naphthylamine (Sigma-Aldrich, St Louis Mo) enabled tracking the efficiency of metabolite recovery between sample extracts; (4) high quality solvents (ACS grade >98% purity) were used; (5) chromatography runs were reproducible with minimal shifts in retention time (typically RSD <0.1 min); (6) sample injection volume was adjusted to result in minimal (<5%) saturated peaks; (7) consistent temperature was maintained in the autosampler and column compartments; (8) simultaneous internal reference mass correction was performed; (9) a clean and stable mass spectrometer ion source was used; (10) complex, unbiased peak finding software was utilized; and (11) public and proprietary databases/libraries were accessed for compound annotation and identification.

### Chemical Standards

Unlabeled, stock solutions of 10 mg/mL inorganic external standards: 9-Anthracene Carboxylic acid and 1-naphthylamine (Sigma-Aldrich, St Louis Mo) were diluted at 1∶10,000 in the injection solvent vials. Cyclic adenosine diphosphate ribose (cADPR) and all other chemical standards were purchased from Sigma-Aldrich (St. Louis, MO). Standard stock solutions of 1 mg/ml in methanol were prepared and diluted for analysis. N1(5-phosphoribosyl)-AMP was synthesized from a cADPR standard by hydrolysis of the cADPR phosphoanhydride linkage [25].

### RP LC/MS

Resuspended aqueous phase sample extracts for all 48 samples were separated by RP chromatography and analyzed by LC-ESI -, LC-ESI+ and APCI +. Although we analyzed the different extracts separately in this study, one can also combine the three sample extracts corresponding to each condition, dramatically reducing the number of samples to be analyzed. Metabolite separation was performed on an Agilent 1200 LC (Agilent Technologies, Santa Clara, CA, USA). RP separation was achieved by injecting 1 µL on a ZORBAX SB-Aq column (2.1×50 mm, 1.8 µm), preceded in series with a ZORBAX -SB-C8 Rapid Resolution Cartridge (2.1×30 mm, 3.5 um). The Agilent 6520 Quadropole Time-of-Flight LC/MS (Q-TOF) was equipped with an electrospray (ESI) ion source or an atmospheric pressure chemical ionization (APCI) ion source (*see Methods S1 for further details*). The organic phase extracts containing a mixture of chloroform and methanol, were centrifuged at 16,000 g, 4°C for 10 min. in order to remove any cellular debris. The supernatants were dried briefly in a SpeedVac, resuspended in LC solvent and analyzed by APCI, primarily for the analysis of neutral molecules, such as lipids, as APCI causes little or no fragmentation of the analyte, and is suitable for both volatile and thermally stable compounds.

### ANP LC/MS

Aqueous Normal Phase (ANP) is a good choice for separation of hydrophilic metabolites on silica hydride columns. Prior sample derivatization is not required, it is compatible with LC/MS, and it exhibits rapid re-equilibration, and reproducible chromatography [17], [18].

Two separate, general purpose ANP chromatographic methods were used to achieve retention and separation of compounds because ammonia was observed to permanently alter the physio-chemical properties of the silica hydride surface. Therefore a separate Cogent Diamond Hydride (150 mm×2.1 mm) column (MicroSolv Technologies, Eatontown, NJ) was dedicated for each of two methods: (1) for neutral and basic compounds, analyzed in (+) ESI mode, and (2) for neutral and acidic compounds analyzed in (−) ESI mode (*See Methods S1 for further details*).

### GC/MS Data Acquisition and Peak Finding

The organic phase extracts for each of the samples were dried and derivatized as previously described [26]: An Agilent 7890 GC was equipped with a 30 m Agilent DB-5MS column. All GC-MS experiments were performed as described in the Agilent user manual [19]. The data was extracted using both AMDIS (NIST) spectral deconvolution, as well as Agilent “Find by Chromatographic Deconvolution” software (Agilent Technologies, Santa Clara, CA). The Agilent-Fiehn GC/MS Metabolomics RTL Library was used for compound identification [27].

### Chemical Entity Definitions

There is currently no standard terminology for what a chemical entity represents along different stages of a metabolomics workflow. In the current study, a “feature” describes a chemical entity that is found as a result of using an unbiased (untargeted) peak finding algorithm such as the “Molecular Feature Extractor” (MFE), or by targeted mining algorithm such as “Find by Formula”. A feature has neither been annotated nor identified (see further details in *Methods S1*). Features are said to be “annotated”, i.e. provisionally identified, when they have an empirical formula assigned to the feature based on a database match. An “annotated metabolite” has an accurate mass within a narrow specified mass tolerance window that matches one or more annotations in an accurate mass database, such as the METLIN database. A corresponding empirical formula is also calculated from the abundance, and a distribution of observed isotopes. The term “metabolite” is used when there is evidence for confirming its identity, for example by accurate mass and retention time matching to a database of standards (AMRT), and/or by MS/MS spectral library matching.

### Data Acquisition and Feature Finding

Untargeted data acquisition on the LC/Q-TOF was performed using Agilent MassHunter™ (“Acquisition”) B.03.01 software (Agilent Technologies, Santa Clara, CA, USA). The data was deconvoluted into individual chemical peaks with Agilent MassHunter™ Qualitative Analysis B.03.01 (MassHunter™ Qual, Agilent Technologies, Santa Clara, CA, USA), using Molecular Feature Extractor (MFE), a naïve or “untargeted” data-mining algorithm, which finds groups of co-variant ions for each unique feature in a chromatogram. Raw data files were processed by aligning the isotopes, dimers and adducts for each calculated neutral mass, having a corresponding RT and abundance.

A targeted, “Find by Molecular Formula” algorithm was used to find features known to be associated with *P. falciparum* using the target list of literature derived,annotated elemental formulas that were derived from the publicly available, MPMP database [12]. This was used to calculate the theoretical accurate mass for each entry and added to a customized, malaria specific, Personal Compound Database and Library (PCDL). This malaria PCDL was uploaded into MassHunter™ Qual software and used for targeted data mining. A series of extracted ion chromatograms (EICs) were saved as *xml* formatted files and were used for subsequent statistical analysis and data visualization in Mass Profiler Professional (MPP) software.

### Data Filtering, Statistical Analysis and Visualization

For LC/MS data files, separate projects were created in MPP; each corresponding to the pH of the extraction solvent, and the ion mode and the polarity of the ESI source. Under each project, a “child” was created, that corresponded to all data files collected for an experiment (for example, all the ESI+ data files for pH2). Three separate projects were created for GC/MS data files. For each experiment, the data files were binned, aligned, and log_2_ transformed and baselined to zero. The features found in each LC/MS and GC/MS data file was filtered based on a relative frequency (Rel Freq) threshold, which corresponds to the number of features that were found in a defined percentage of all biological sample replicates, in at least one or more conditions. For example, features that were present in all (100%) of the replicates, i.e., 100% Rel Freq threshold, were additionally filtered based on an absolute coefficient of variation of less than 100% (CV<100%) within a particular condition. Separate, filtered lists of features for each condition were created for use in all subsequent downstream data analyses. Unsupervised principal component analysis (PCA) was performed with mean centering and scaling to display the inherent variance between the metabolic phenotypes. Infection state, pH, SLO treatment, or combinations of these variables were used for distinguishing between conditions.

Statistical evaluation of the data was performed using univariate and multivariate analyses, including the Welch’s unpaired *t-*test for independent pairs of groups, and one way ANOVA for multiple groups. The effects of infection state and extraction solvent pH on classifying the samples were compared. A cutoff value of *p*<0.05 was considered statistically significant in one-way ANOVA, using the Benjamini and Hochberg False Discovery Rate set to 5% for multiple testing corrections [28]. The difference between each pair of means with appropriate adjustment for multiple testing in the ANOVA was investigated with the Tukey multiple comparison test [29]. This resulted in a matrix table, revealing the results for each pair of conditions as a *p-*value. Parameters were selected that computed the Fold Change and *p-*values.

### Identification

While high mass accuracy (<5 ppm) alone is insufficient to exclude enough candidates with complex elemental compositions (C, H, N, S, O, P, and potentially F, Cl, Br and Si), for unambiguous identification, our use of isotopic abundance pattern information as a further constraint removed >95% of false candidates [30]. For LC/MS data, the annotated metabolite compound identifiers were generated based on accurate mass, isotope ratios, abundances and spacing, as well as RT and MS/MS spectral matching to standards for select compounds. The metabolites found to have interesting pathway mapping characteristics, or to be significantly different between IRBCs and NRBCs, were identified by accurate mass matching, and/or with orthogonal RT matching to the METLIN database using the Agilent Identification Browser (ID Browser). The results were sorted and a single annotation with a matching elemental formula from METLIN was selected. The Agilent METLIN database (B.04) contained over 25,000 compounds, with links to KEGG identifiers, CAS numbers, HMDB and LIPID MAPS identifiers. From this, an inclusion list of *m/z* and RT values was created in MPP. The lists of annotated, differential metabolites were used as target masses for subsequent confirmation of metabolite identity by accurate mass MS/MS and spectral library matching. Several NRBC and IRBC samples were re-analyzed by targeted MS/MS analysis on the Q-TOF. MS/MS spectra were acquired at three collision energies (10, 20 and 40 eV) and the sample spectra were compared and matched to a library of standard spectra in METLIN PCDL, generated from MS/MS spectra of approximately 2000 standards.

### Databases

A list of publicly available databases that were used in this study is provided in *Methods S1*.

### Pathways

Pathway analysis was performed using Mass Profiler Professional (MPP) v12.5, including the Pathways add-on in Pathway Architect. The *P. falciparum* 3D7 strain of pathways in.*owl* format were uploaded from BioCyc and queried under the Pathway Analysis feature to generate matching pathways that were graphically rendered by the Pathway Architect engine. In addition, HeatStrips were generated that summarized a separate HeatMap table of results for identified metabolites in NRBC and IRBC conditions. The annotated metabolite IDs from our annotated list of results were mapped to IDs in the underlying BioCyc database using open source BridgeDB software [31]. The results revealed a table summary for the total number of metabolites per pathway and the number of matching metabolites. Each pathway was then analyzed for the number of possible associations, resulting in a sorted list of pathways that facilitated data interpretation.

## Results

### Comprehensive Metabolite Extraction and Separation Strategies Facilitate Detection of a Larger Proportion of the Parasite Infected RBC Metabolome

An untargeted profiling strategy was employed to collect, analyze, and identify metabolites and conduct pathway analyses (**Figure S1**). Due to the broad spectrum of physicochemical properties inherent to metabolites, sample preparation and detection methods need to be extensive. Protein precipitation as well as sub-micron filtering of extracts facilitated highly reproducible chromatographic retention times for each metabolite. The net result was a large number of chemical features for data collected by RP LC/MS or ANP LC/MS **(** **Table 1** **)**. The pH of the extraction solvents, chromatography conditions, MS ion modes, and ion polarities all impacted the results significantly.

**Table 1:** Summary of results for the number of features found using MFE, an “untargeted” feature finding algorithm.

Condition	pH2	pH7	pH9	pH2	pH7	pH9	pH2	pH7	pH9
	ESI−	ESI−	ESI−	ESI+	ESI+	ESI+	APCI +	APCI +	APCI +
NRBC	1433	915	1267	2029	1401	2626	1128	463	500
IRBC	1327	1151	1232	2042	1542	2661	665	460	652
*Rel Freq. 75%	1660	1282 (245)	1412 (273)	2175 (602)	1838 (602)	2822 (650)	916	502	679
*Rel Freq. 100%	1259	917 (124)	1086 (178)	1550 (425)	1247 (556)	2086 (378)	486	342	483
CV<100%	781	547 (106)	649 (161)	936 (376)	662 (387)	1083 (350)	284	235	271

The numbers in parentheses () denote the number of features found by LC/MS using ANP chromatography. Data was not collected for APCI+mode and for pH 2 ANP/ESI−.

* The % relative frequency (Rel Freq.) corresponds to the number of features found in 75% or in 100% of all biological sample replicates, and in at least one or more conditions. Only features with a coefficient of variation less than 100% (CV<100%) were used for subsequent significance testing.

For RP LC/MS analyses, over two thousand features were detected in ESI+mode; between 900–1500 in ESI - mode; and several hundred features were detected in APCI+mode (**Table 1**). The overall trend in the total number of features for the different ionization/polarity modes was ESI+> ESI−>APCI+. The number of features detected by ANP LC/MS (**Table 1**, results in parentheses) was approximately one/third of that detected by RP separation.

PCA analyses for LC/MS results (data not shown) indicated that the pH of the extraction solvent was the largest single contributor to the variance observed between sample groups, and corroborated our previously published findings [20]. The extraction solvent pH, as well as the type of column used: RP or silica hydride column (ANP mode), both had an impact on the number of differential features with FC >2 and P<0.05 (**Table S1**). Pair-wise *t-test*s between IRBC and NRBC sample groups revealed 115 differential features from pH 7 solvent extracts that were detected by RP/ESI+, and as few as 7 differential features in pH 9 solvent extracts detected by ANP/ESI+. These results were significant even when accounting for the False Discovery Rate (FDR). Fewer features, about a third, were detected by APCI+compared to ESI+, probably due to its lower range of *m/z* of detection (≤ *m/z = *300) and the nature of metabolites that can be detected by this ionization mode.

In summary, regardless of prior data filtering steps, pH 2 and pH 9 extracts recovered more total features than pH 7 extracts. However, the pH 7 extracted samples were the most discriminatory between NRBC and IRBCs, with more significantly differential features (*p<0.05*) detected than at pH 2 or pH 9. When differential features were annotated, some of the compounds were found at more than one pH (**Figure S2**). However, a significant proportion of compounds were unique to each extraction pH, illustrating the importance of extraction under all three pH conditions to maximize metabolite recovery.

### Unsupervised Analyses of Metabolite Profiles Differentiate RBC Infection States

After acquiring and filtering the global metabolite content, we used the filtered entity lists (CV <100%) in unsupervised PCA to classify samples belonging to NRBC or IRBC (**Figure S3A and S3B**). PCA analysis suggested a subset of annotated compounds that could be classified as differentiators of the infected state. PCA of LC/MS data acquired in ESI+ and ESI− modes for pH 7 solvent extracted samples clearly differentiated sample groups based on RBC infection state. For a separate set of samples where NRBCs and IRBCs were selectively permeabilized by SLO pre-treatment prior to extraction, only a marginal SLO effect was observed that was insufficient for classifying the samples (data not shown). Therefore, to increase the pool size for NRBC and IRBC groups, the SLO-pre-treated data was combined with SLO-untreated data, improving the statistical power for differentiating the two infection state conditions.

### Provisional Metabolite Annotation of Untargeted, Discovery Based Analysis

METLIN database annotation resulted in 171 putatively annotated metabolites (subsequently referred to as “annotated metabolites”), encompassing non-polar, polar and volatile compounds. A complete list of annotated metabolites, resulting from MFE and FbF peak finding algorithms for RP/LC-MS data, is shown in **Table S2**. 104 annotated metabolites were found and over half had a METLIN database match to one or more compounds (i.e. isomers). After accounting for compound redundancies, approximately one quarter of annotated metabolites revealed a single match to METLIN content, while a fifth of the total had two annotated matches in the METLIN database. ANP/LC-MS analysis found 52 annotated metabolites using MFE analysis (**Table S3**), and 48 annotated metabolites were matched using FbF, a targeted mining method based on a database of empirical formulas. In addition, the identity of a subset of these annotated metabolites was confirmed by Q-TOF MS/MS and/or retention time (RT) matching to chemical standards (**Table S4**).

**Figure 1:**
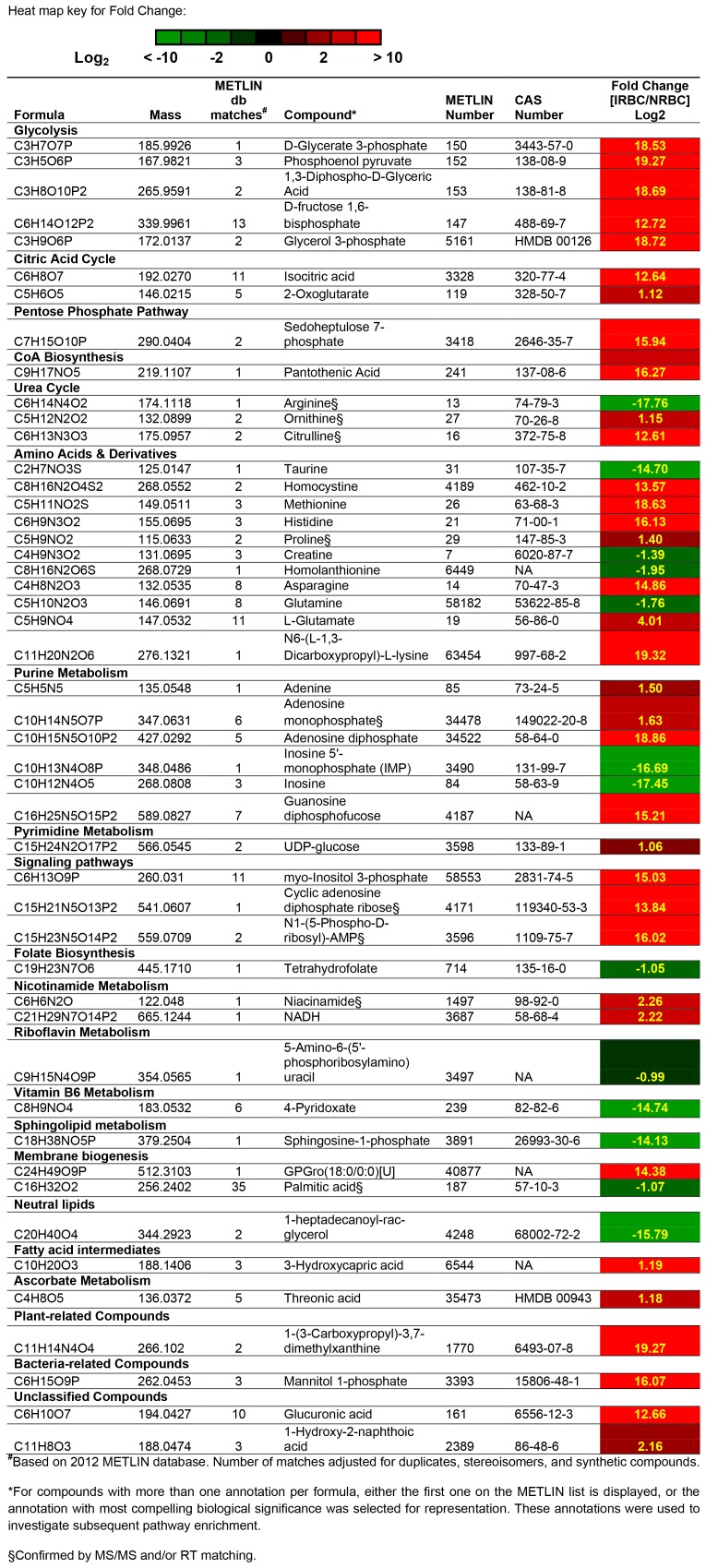
Features detected by global untargeted analysis and inferred from METLIN database searches having an absolute fold change |[IRBC/NRBC] Log2| ≥1.0. A complete listing of all features detected by global untargeted analysis is presented in Table ST1. Compounds confirmed by MS/MS and/or RT matching are indicated with a “§” symbol.

Highly differential metabolites with an absolute Log_2_ ratio of one or greater (│[IRBC/NRBC] Log_2_│≥1, *p<0.05*) from both datasets and their associated biological pathways are summarized in **Figure 1**. Among the metabolites identified by untargeted global profiling with a two-fold or greater change (**Figure 1**) were several glycolytic intermediates, components of the citric acid (TCA) cycle and pentose phosphate pathways, the urea cycle, amino acids and their derivatives. Multiple compounds associated with purine metabolism were highly differential while only one intermediate of pyrimidine metabolism was differentially expressed. Also highly differential were metabolites associated with CoA, lipid, nicotinamide, ascorbate, folate, and vitamin B6 biosynthesis and metabolism.

Arginine was either undetectable or detected at very low levels in IRBC samples with IRBC/NRBC Log_2_ FC = −3.95. Similarly, inosine and inosine 5′-monophosphate (IMP), a key purine metabolism intermediate, were depleted in IRBC samples. The identities of urea cycle intermediates, ornithine and citrulline, were also confirmed and found to be elevated in IRBCs. The results for arginine and the amino acid derivatives, citrulline and ornithine, obtained using quadrupole time-of-flight (Q-TOF) mass spectrometry in this study are consistent with previously reported, quantitative MS/MS analyses using triple quadrupole mass spectrometry [32], [33].

Novel, differentially expressed, annotated metabolites that were detected in this study included (1) GPGro (18∶0/0∶0)[U], a glycerol phosphoglycerol associated with membrane biogenesis in other organisms; (2) 1-(3-Carboxypropyl)-3,7-dimethylxanthine, previously detected as a metabolite of pentoxifylline, and representative of plant-derived methylated xanthines such as theophylline and caffeine; (3) guanosine diphosphofucose and mannitol 1-phosphate, intermediates of fructose and mannose metabolism, amino sugar and nucleotide sugar metabolism; (4) 1-Hydroxy-2-naphthoic acid, a compound found in bacteria associated with polycyclic aromatic hydrocarbon degradation; and (5) two related ribonucleoside phosphates, potentially associated with *Plasmodium* signaling: cyclic adenosine diphosphate ribose and N1-(5-Phospho-D-ribosyl)-AMP. These two ribonucleoside phosphates are discussed in greater detail below. A few compounds generally associated with plants or bacteria were also highly differential, along with some unclassified compounds.

Lipids were highly represented in our dataset and included a diverse array of compounds including dicarboxylic acids, unsaturated fatty acids, straight chain fatty acids, branched fatty acids, oxo fatty acids, amino fatty acids, octadecanoids, monoacylglycerols, glycerophosphocholines, and sphingolipids (**Table S2**). These results reflect highly active *de novo* fatty-acid synthesis and fatty acid importation into the *P. falciparum-* infected erythrocyte. Of particular note was the very high level of the glycerophosphoglycerol GPGro (18∶0/0∶0) detected in IRBC only. Sphingosine-1-phosphate and the neutral lipid 1-heptadecanoyl-rac-glycerol were greatly depleted in IRBCs.

### Targeted Data Mining of Compounds Associated with *P. falciparum* Infection

A separate, targeted data mining workflow was developed to complement the untargeted, profiling approach. For this workflow, the processed data files were queried with compound formulas that are specifically associated with an organism’s metabolic pathways. A Plasmodium-specific Personal Compound Database (PCD) was constructed from 350 compounds derived from the Malaria Parasite Metabolic Pathways (MPMP) database [12]. Elemental compound formulas were used to generate Extracted Ion Chromatograms (EICs) from the data, which resulted in 72 matches to the database.

**Figure 2:**
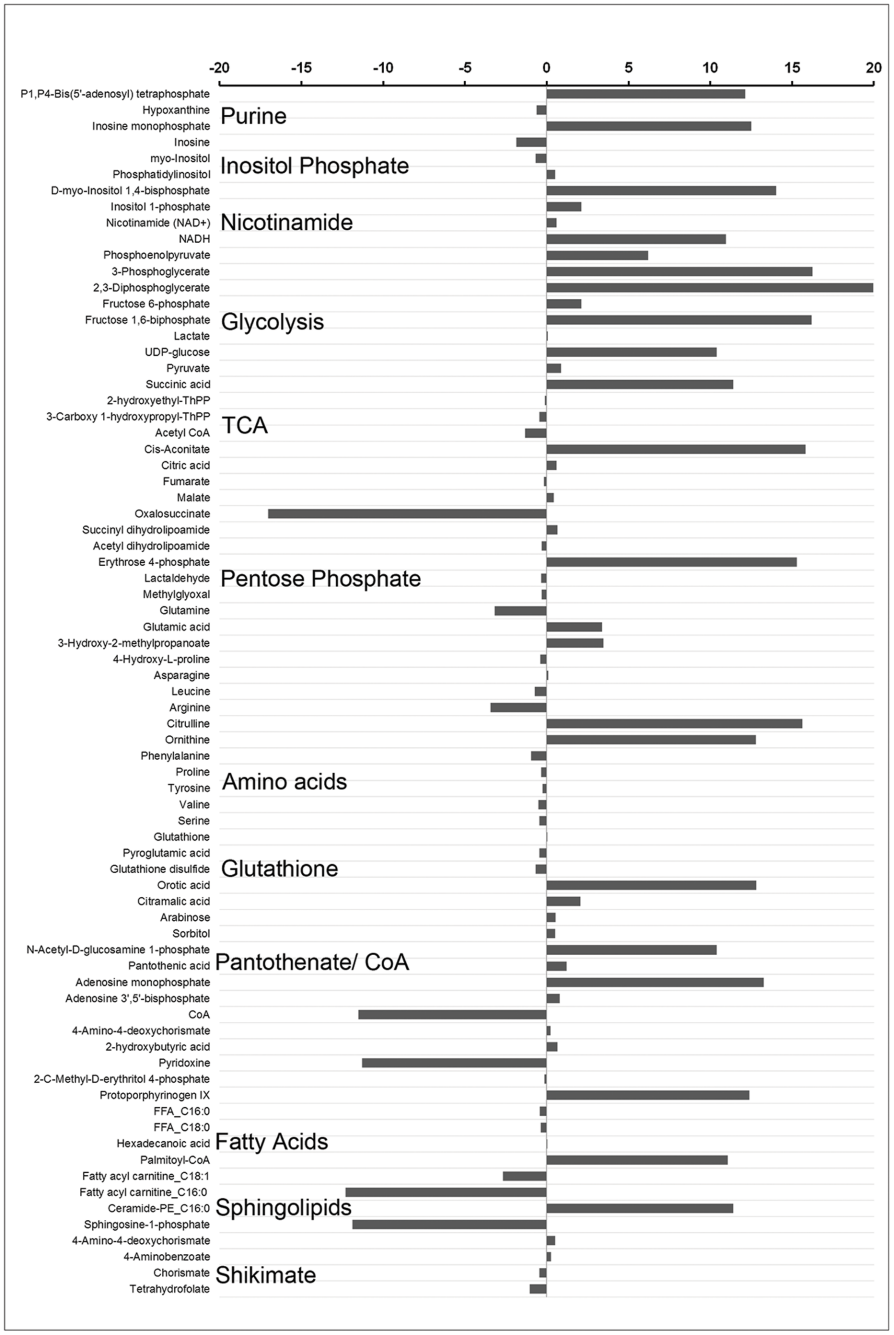
A partial list of identified compounds resulting from “targeted” analysis, showing differential abundances between IRBC and NRBC. The relative abundances are based on IRBC/NRBC Log_2_ ratios for each entity that were significantly (*P*<0.05) different. Also indicated are the biological pathways associated with some of these compounds.

A list of metabolites with MPMP database matches, as well as their [IRBC/NRBC] Log_2_ ratios is summarized in **Figure 2**. The results for several metabolites found to be differentially abundant in IRBCs were consistent with our untargeted data mining approach. This included the glycolytic pathway and compounds associated with purine and nicotinamide metabolism. However, some of the metabolites identified by the targeted data mining strategy had not been detected by the untargeted approach, including erythrose 4-phosphate (pentose phosphate pathway), succinic acid (TCA cycle), palmitoyl-CoA, ceramide-phosphatidyl ethanolamine (PE), and several inositol phosphate metabolic intermediates. Fatty acyl carnitines and sphingosine-1-phosphate levels were observed to be significantly reduced in IRBC as compared to NRBC samples.

### Major Metabolite Super Classes and Metabolic Pathways of *P. falciparum*-infected RBCs

**Figure 3:**
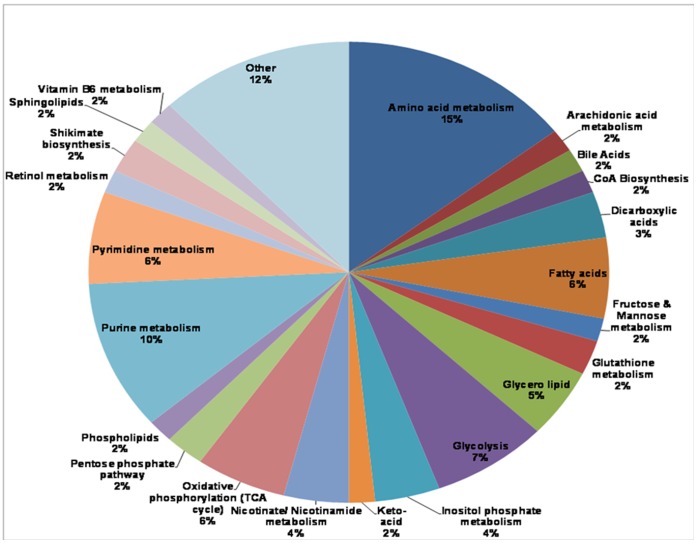
Pie Chart summarizing the distribution and super class (from HMDB) information for IRBC and NRBC metabolites, detected by “untargeted” and “targeted” data mining analyses.

A local database of malaria-associated metabolites was created from the compounds in the MPMP database [12]. Based on results from RP LC/MS analyses, a total of 139 compounds in the database matched across our data files, corresponding to 124 compounds in IRBCs, and 108 compounds in NRBCs. The super class distribution of differentially expressed metabolites is summarized in a pie chart to illustrate the full range of metabolites detected by untargeted and targeted analyses in IRBC and NRBCs (**Figure 3**). Super class annotations for all compounds were based on the HMDB chemical taxonomy [34]. The compounds fell into 22 super classes, covering a wide range of metabolites with different physiochemical properties. Amino acids, purines, pyrimidines and glycolytic intermediates reflected the largest percentage of differential compounds affected by parasite infection. In addition, oxidative phosphorylation, fatty acid, glycerol lipid and inositol phosphate metabolism were all affected.

### pRib-AMP, a Product of cADPR Phosphohydrolysis, is Associated with IRBC Metabolism

**Figure 4:**
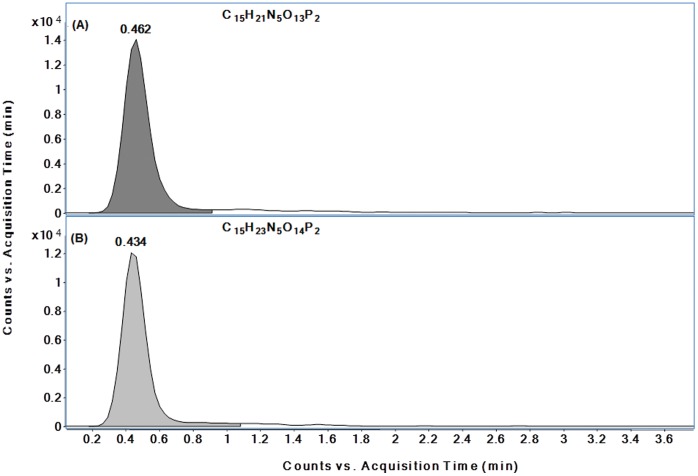
Extracted Ion Chromatograms (EICs) for a derivitized standard mixture containing both (A) cADPR and (B) pRib-AMP, analyzed by LC/MS ESI (−) mode.

The utility of untargeted analysis as a discovery approach was highlighted by the identification of metabolites corresponding to elemental formulas C15H21N5O13P2 and C15H23N5O14P2, which were elevated in IRBC samples relative to NRBC. The identities of the compounds were subsequently confirmed to be cyclic adenosine diphosphate ribose (cADPR), and N1-(5-phospho-D-ribosyl)-AMP (pRib-AMP) or acyclic ADPR, respectively. cADPR is a second messenger involved in the regulation of cellular Ca2+ homeostasis in eukaryotic cells [35] and has also been implicated in Plasmodium signaling [36]. pRib-AMP has recently been shown to be a cADPR phosphohydrolysis product in mammalian cells via a Mn^2+^-dependent ADP ribose/CDP-alcohol pyrophosphatase [25], raising the possibility that a similar reaction could occur in *P. falciparum*. LC/MS analysis of a cADPR standard revealed an EIC peak for m/z = 540.0538, with m/z and RT similar to those observed in the samples. Since a commercially available standard for pRib-AMP was not available, it was generated by chemical hydrolysis of the cADPR standard that partially converts it to the derivatized product, pRib-AMP [25]. Based on an EIC for m/z = 558.0644, corresponding to C15H23N5O14P2, the derivatized solution contained an approximately equimolar mixture of cADPR (**Figure 4A**) and pRib-AMP (**Figure 4B**). The LC/MS spectral scan results based on EICs and isotope distributions of the cADPR standard and NRBC sample (**Figure S4 A and B**), as well as the pRib-AMP standard and IRBC sample (**Figure S4 C and D**) strongly suggested that the compounds in the biological samples corresponded to these standards.

**Figure 5:**
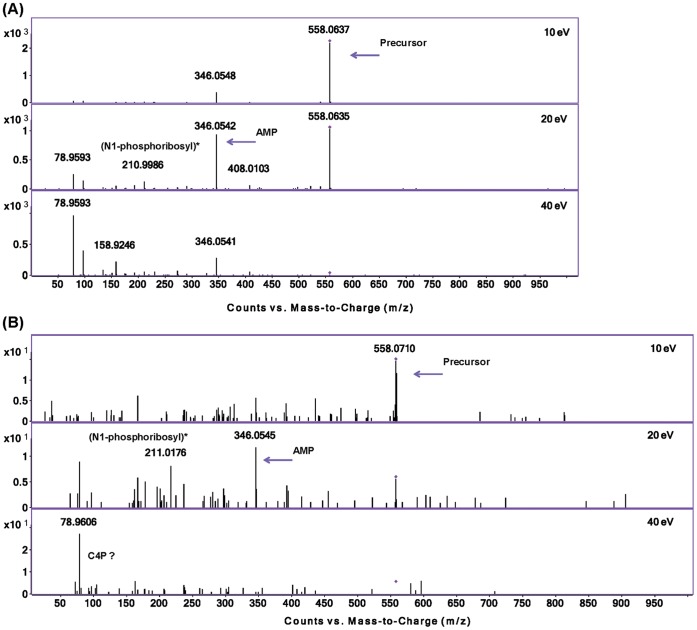
Extracted MS/MS spectra for *m/z* 558.0644, detected in ESI (−) mode at three collision energies: 10, 20, 40 eV. The empirical formula corresponding to the neutral mass, C_15_H_23_N_5_O_14_P_2_ corresponds to a compound with a single database entry in METLIN and annotated as N1-(5-phospho-D-ribosyl)-AMP (pRib-AMP). MS/MS spectra were collected for this compound from: (**A**) the chemically derivitized cADPR product, pRib-AMP, and, (**B**) an IRBC sample.

Q-TOF MS/MS analysis was performed at three collision energies (10, 20, 40 eV), followed by spectral analysis of the cADPR standard and its derivatized analogue, pRib-AMP. The fragmentation patterns of the precursor ion, *m/z* 558.0644 revealed ions consistent with AMP and N1-phosphoribosyl fragments of pRib-AMP (**Figure 5A**). Subsequent extracted MS/MS spectral comparisons of the *m/z* 558.0644 precursor from an IRBC sample (**Figure 5B**) confirmed the production of a similar pattern of predominant fragment ions.

**Figure 6:**
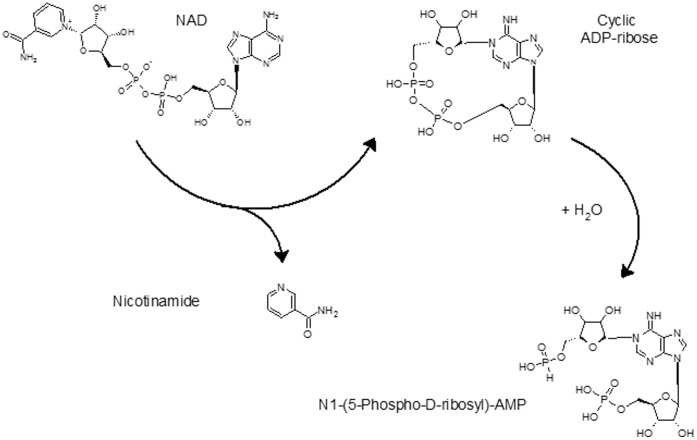
A proposed enzymatic pathway for generating pRib-AMP from cADPR in *P. falciparum.*

In mammalian cells, pRib-AMP is an intermediate in histidine biosynthesis, and can also be produced as a result of enzymatic cADPR hydrolysis [25]. Although the presence of pRib-AMP has not been previously reported in *P. falciparum*-infected erythrocytes, the concurrent elevation of cADPR, pRib-AMP, and nicotinamide in these cells suggest the possibility that this hydrolytic pathway may be utilized by *P. falciparum*-infected erythrocytes (**Figure 6**).

### Arginine Depletion in IRBC

Arginine is efficiently converted to ornithine by the malaria parasite, resulting in arginine depletion both in vitro and in vivo [37]. Only two enzymes of this cycle have been previously described for *P. falciparum*: carbamoyl-phosphate synthase (6.3.4.16: PF3D7_1308200); and Arginase (3.5.3.1: PF3D7_0906500). In our study, greatly reduced levels of arginine (log_2_ ratio = −17.76) were detected in IRBC relative to NRBC (**Figure 1**). Previously published, quantitative LC/QQQ analyses of arginine, ornithine and citrulline, show consistency with our untargeted findings [38]. The depletion of arginine in IRBC relative to NRBC samples was accompanied by an increase in ornithine as well as citrulline levels, suggestive of a functional urea cycle in *P. falciparum* or production of citrulline from arginine by a nitric-oxide synthase. These findings illustrate the point that the metabolomics approach may enable the identification of pathways that cannot be found by gene homology searches alone.

### Glycolysis and Branched TCA Metabolites

Carbohydrate metabolism has been extensively studied in *P. falciparum* and certain unique features of central carbon metabolism for this parasite have been noted [39], [40]. Due to its dependence on glucose fermentation for energy [41], the blood-stage malaria parasite is thought to operate an efficient, yet much less pronounced central carbon metabolic system. Inclusion of metabolites related to glycolysis and the TCA cycle in our global analysis served to confirm the validity of this approach to detect multiple components of a metabolic pathway during *P. falciparum* intra-erythrocytic development.

**Figure 7:**
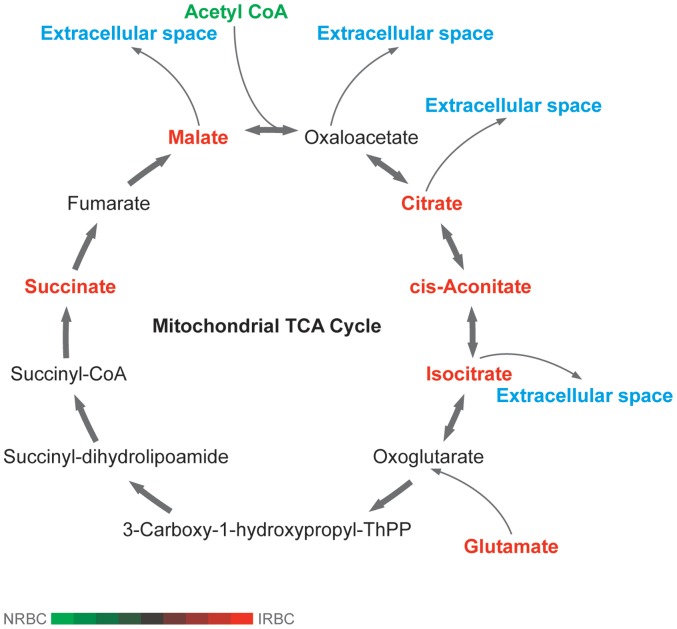
Untargeted, global LC/MS metabolomics analysis for *P. falciparum* infected RBC cultures reveal metabolites associated with the mitochondrial TCA cycle. Differential and quantified metabolites are shaded in red.

As a result of its dependence on glycolysis, Plasmodium has a dampened TCA cycle that is largely dissociated from glycolysis [40]. Using our PCD of targeted list of compounds, we searched for several previously identified metabolites that have been associated with the “branched TCA cycle” **(** **Figure 7**) unique to Plasmodium [39]. TCA-related metabolites identified in this way were: Acetyl CoA (high in NRBC), glutamate, succinate, malate, citrate/isocitrate, and aconitate (high in IRBC), and succinyl-dihydrolipoamide (similar in IRBC & NRBC). GC/MS analysis detected several hexoses in the samples, including fructose, galactose (only in IRBC), allose, talose and tagatose (only in NRBC) (**Table S5)**. Whereas glucose was almost four-fold depleted in IRBCs, pyruvic acid and lactic acid levels were similar between NRBC and IRBCs, consistent with the conversion of pyruvate into lactic acid and efficient export of lactic acid from the parasite and infected erythrocyte [42].

### Global Profiling Results for Annotated Metabolites and *P. falciparum* Pathway Mapping

Biological pathway analyses provide a powerful medium with which to explore and reduce the complexity of large datasets. Pathway gene enrichment analysis [43] based on transcriptomics data has previously been shown to be a powerful means for identifying biochemically active pathways [44]. Utilization of the Pathway Architect software enabled us to organize numerous metabolites into canonical pathways. Compared to genes, primary metabolites represent a relatively sparse matrix. In untargeted profiling experiments, it is possible to have multiple chemical formulas that can correspond to the same measured mass. Since most metabolic pathways contain one or more metabolites and corresponding chemical formulas that are unique to that metabolic pathway [45], *in silico* testing of annotated metabolite enrichment can be evaluated as a surrogate for experimental identification of metabolites. Therefore, the metabolite IDs from the list of IRBC metabolites (**Tables S2 and S3**) were queried against the computationally derived set of pathways for the 3D7 isolate of *Plasmodium falciparum* (BioCyc, [46]). To investigate global differences between NRBCs and IRBCs using this pathway approach, a BioPax formatted file of the Plasmodium pathways was loaded into the Pathway Architect module and queried. This generated a list of known pathways in the software, which were most strongly represented by the input metabolite list (**Figure S5**). Over 50 distinct pathways with at least one matching entity to BioCyc content were observed. A number of pathways were impacted or apparently enriched; most prominently the superpathway for arginine and polyamine biosynthesis, intermediates of glycolysis, the TCA cycle and nucleotide metabolism. For processes involving energy production such as glycolysis, almost all of the compounds matched the annotated metabolite list, revealing significantly higher enrichment in IRBCs. While many of these pathways were noted previously during visual inspection of the metabolite list, some had not been readily discernible, such as the enrichment of 11/46 components of the chorismate superpathway (data not shown). This pathway overlaps with the shikimate pathway that is present in apicomplexan parasites but absent in animals [47].

## Discussion

This study explored a comprehensive untargeted metabolomics workflow for *Plasmodium falciparum*-infected host erythrocytes in cell culture that has not been previously described. The availability of accurate mass metabolite databases, a database of computationally derived list of compounds for *Plasmodium falciparum*, spectral libraries and pathway maps were critical to this approach and a similar strategy may be applied to other infections and disease states.

Previous studies in our laboratory examined a variety of factors that contribute to optimization of the analysis of the erythrocyte metabolome [16] and this served as the basis for the methods used in this study. Parameters that were examined previously included extraction solvent composition, mobile phase composition, and extraction solvent pH. By maximizing detection coverage for non-polar, polar and volatile compounds in our study, we increased the probability of matching a subset to annotated metabolite databases. This was a critical point, since a sparser dataset relying on only a single extraction solvent and ion/polarity for MS detection would have yielded less compelling results. Over a thousand features were detected across all conditions, and over 150 non-redundant compounds were identified. The combined results for untargeted and targeted metabolite mining were assigned to a total of 22 metabolite super classes, the largest of which were related to amino acid metabolism, nucleic acid metabolism, and glycolysis. While data acquisition and analysis in an untargeted mode was valuable for detecting a wider range and larger number of compounds, targeted data mining for known compounds derived from the *Plasmodium* literature using the Find by Formula algorithm was more selective and sensitive. Thus, these two approaches were complementary for global profiling of the *P. falciparum*-infected erythrocyte metabolome.

Previously-defined *P. falciparum* metabolic pathways and their components established a framework for applying global LC/MS based metabolomics to explore this complex host:parasite relationship. Examples of matches using our inferential pathway analysis included carbohydrate metabolism (glycolysis, pentose phosphate cycle), purine metabolism, CoA biosynthesis, inositol phosphate metabolism, nicotinamide metabolism, amino acid metabolism, glycerophospholipid metabolism, and sphingolipid metabolism. Strikingly, glycolytic pathway intermediates were the highest abundance species detected in IRBC. In addition, although one component of the shikimate pathway had been identified through our targeted data mining approach, a larger number of compounds associated with the related chorismate superpathway were identified by pathway analysis of untargeted data.

In addition to glycolysis, the TCA cycle has been well-studied in *P. falciparum*. Although some compounds in this cycle may have been at low and therefore undetectable levels, we were able to detect several intermediates that are known to be retained in the parasite mitochondrion in IRBC: 2-oxoglutarate, S-succinyl dihydrolipoamide, succinate, and fumarate [39]. In contrast, intermediates known to be secreted by the parasite (malate, isocitrate, cis-aconitate, citrate, and oxaloacetate) [39] either were undetected or were not enhanced in IRBC relative to NRBC. The reciprocal relationship observed between glutamine and glutamate in this study is consistent with the efficient importation of glutamine by the *P. falciparum*-infected erythrocyte [48] and its conversion in the oxidative branch of the TCA cycle to glutamate and 2-oxoglutarate by glutamate dehydrogenase [39].

Along with accurate mass MS/MS confirmation, the identities of arginine, citrulline and ornithine were independently validated and quantified by LC-QQQ mass spectrometry [38]. The results confirmed depletion of arginine, along with enhancement of ornithine in infected erythrocytes, consistent with Olszewki et al. [37]. In our study, however, citrulline levels were also elevated in infected erythrocytes. Citrulline can be generated from L-ornithine by ornithine carbamoyltransferase as part of the urea cycle, or from L-arginine by arginine deaminase, or nitric oxide synthase. These observations suggest that *Plasmodium* may possess one or more enzymes for citrulline production not previously described for this genus, resulting in a nearly-complete urea cycle.

Purines, particularly hypoxanthine, are essential nutrients that the intracellular malaria parasite requires for growth and multiplication. Salvaging purines from the host milieu is an important physiological requirement for growth and multiplication of *P. falciparum*. We detected several purine metabolites including adenine, adenosine monophosphate, adenosine diphosphate, guanosine diphosphofucose as well as P1,P4-Bis(5′-adenosyl) tetraphosphate. Hypoxanthine was detected in all erythrocyte extracts, but was not significantly elevated in IRBCs, indicating that this compound is rapidly consumed during purine biosynthesis.

Depletion of Inosine 5′-monophosphate (IMP) was observed in IRBCs but not in NRBCs. IMP is a key component of purine biosynthesis, and may be used in the *Plasmodium* purine salvage pathway to produce adenylate or guanylate nucleotides [49]. IMP depletion in IRBCs was accompanied by increases of inositol phosphate and D-myo-inositol 1,4-bisphosphate. This pathway ultimately provides inositol intermediates required for glycosylphosphatidylinositol anchor biosynthesis, a key post-translational modification of *Plasmodium* membrane proteins [50].

Several compounds were differentially expressed which have not previously been associated with *P. falciparum* metabolism. Some of these compounds are associated with metabolic pathways previously identified in plants and bacteria and may serve as targets for drug development. One novel finding based on our discovery approach was the differential elevation of pRib-AMP and cADPR levels in IRBCs. Both cADPR and pRib-AMP are adenine-containing nucleotides that have been studied primarily in mammalian systems. cADPR is a naturally occurring metabolite of nicotinamide adenine dinucleotide (NAD+), and is an important signaling molecule that mobilizes endogenous Ca2+ from non-mitochondrial stores in a variety of mammalian and invertebrate tissues [35], [51]–[53]. cADPR is of particular interest in malaria because it is known to regulate the release of intracellular Ca2+ during *P. falciparum* erythrocyte invasion [54] and to be more effective than and to work independently of, inositol 1,4,5-trisphosphate in controlling Ca2+ release in *P. falciparum-infected* erythrocytes [55]. Differential expression of both cADPR and pRib-AMP in infected erythrocytes provides independent support for the role of cADPR in *Plasmodium* blood stages and raises the possibility of a novel mechanism for its turnover in parasite-infected cells. Since Ca2+ signaling controls a critical step in malaria parasite invasion, components of the cADPR signaling pathway, as well as mechanisms for its cellular turnover that may entail its conversion to pRib-AMP are of biological and pharmacological interest.

In summary, this study illustrates the value of LC/MS profiling for acquiring and querying metabolomics data of *P. falciparum*-infected erythrocytes in an untargeted, discovery-driven approach. While this manuscript was in review, Lakshmanan et al published an untargeted LC/MS study [56], to show evidence for intermediates of a plant-like α-linoleic acid pathway in patient plasma and in vitro cultured RBCs. Although their strategy was less comprehensive and yielded fewer total metabolites than we report here, it underscores the significance of the untargeted approach in discovery metabolomics. It should be noted that among the metabolites detected in our study were several of the α-linoleic acid pathway metabolites described in their study, including methyl jasmonate and traumatic acid (dodecanedioic acid). While these metabolites were differentially expressed in IRBCs, they were not as highly differential as some of the other compounds examined.

Screening of the *Plasmodium* genome for orthologous genes with sequence homology to defined enzymes for several of these pathways in other species or alternative approaches for identification of analogous genes will be an important next step. Techniques such as activity-based metabolomic profiling [57] may subsequently be used to determine whether *Plasmodium* gene orthologs possess the expected enzymatic function. It should be noted that 50% of the chemical features detected could not be matched to the existing METLIN database, and alternative strategies for identification of these compounds are needed. This study shows the tremendous potential of a global mass spectrometry-based platform to expand the universe of defined *Plasmodium* metabolites and identify potentially novel metabolic pathways of this important human pathogen.

## Supporting Information

Figure S1 **Untargeted profiling workflow for LC/MS and GC/MS separation, detection, processing, and annotation of data which can then be used for mapping onto metabolic pathways.**
(TIF)

Figure S2 **Venn diagram of the distribution of differential annotated features between NRBC and IRBC groups for pH 2, 7 and 9 extraction conditions (** ***P<*** **0.05).**
(TIF)

Figure S3 **(A) 2D PCA plot of pH 7 solvent extracts for 662 metabolites analyzed in ESI positive ion mode and (B) 547 metabolites analyzed in ESI negative ion mode (See** **Table 1** **).** IRBC: red filled squares, 0 SLO; red filled triangles 250 U SLO; NRBC: blue filled squares, 0 SLO; blue filled triangles 250 U SLO.
(TIF)

Figure S4 **LC/MS ESI (−) extracted spectral scan comparisons for cADPR and pRib-AMP.** (A) cADPR: an incomplete *in vitro* chemical derivitization reaction for cADPR or C_15_H_21_N_5_O_13_P_2_ (*m/z* 540.0536); (B) cADPR in a NRBC sample; and for (C) pRib-AMP or C_15_H_23_N_5_O_14_P_2_: the extracted spectrum for the derivitized product (*m/z* 558.0649); (D) extracted spectrum for pRib-AMP in an IRBC sample. Black lines are instrument measured isotopes. Height and spacing between the red boxes correspond to the theoretical, MFG calculated isotopes for each empirical formula.
(TIF)

Figure S5 **Pathway analysis of the annotated metabolites for P. falciparum infected RBC cultures was based on querying BioCyc pathways for** ***P. falciparum*** **3D7 strain in Mass Profiler Professional software.** The representative figure and tables are an example, depicting the pathway network and compound matches associated with the superpathway for glycolysis, pyruvate dehydrogenase, TCA and glyoxylate bypass.
(TIF)

Table S1 **Summary of results for differentially-expressed features based on Fold Change (FC) and t-test comparisons between NRBC and IRBC groups.**
(DOCX)

Table S2 **Untargeted and targeted mining results were combined and used for differential analysis of metabolite data acquired by global untargeted analysis using RP/LC-ESI and APCI.**
(DOCX)

Table S3 **Differential analysis for metabolite data acquired by global untargeted analysis using ANP/LC-ESI.** The results for untargeted (MFE) and targeted (FbF) data mining strategies are shown.
(DOCX)

Table S4 **List of metabolites confirmed by MS/MS spectral matching and/or retention time (RT) matching to chemical standards.**
(DOCX)

Table S5 **List of metabolites confirmed by Agilent Fiehn GC/MS Metabolomics RTL Library.**
(DOCX)

Methods S1 **Supplementary Materials and Methods.**
(DOCX)

## References

[1] Programme WGM (2010) World Malaria Report 2010. Geneva: World Health Organization.

[2] Phyo AP, Nkhoma S, Stepniewska K, Ashley EA, Nair S, et al.. (2012) Emergence of artemisinin-resistant malaria on the western border of Thailand: a longitudinal study. Lancet. 10.1016/S0140-6736(12)60484-X PMC3525980 22484134

[3] Bloland PB (2001) Drug resistance in malaria. World Health Organization. WHO/CDS/CSR/DRS/2001.4 WHO/CDS/CSR/DRS/2001.4. 1–27 p.

[4] Oliver SG (1996) From DNA sequence to biological function. Nature 379: 597–600. 8628394 10.1038/379597a0

[5] Fiehn O, Kopka J, Dormann P, Altmann T, Trethewey RN, et al (2000) Metabolite profiling for plant functional genomics. Nat Biotechnol 18: 1157–1161. 11062433 10.1038/81137

[6] Goodacre R (2007) Metabolomics of a superorganism. J Nutr 137: 259S–266S. 17182837 10.1093/jn/137.1.259S

[7] Sana TR, Roark JC, Li X, Waddell K, Fischer SM (2008) Molecular formula and METLIN Personal Metabolite Database matching applied to the identification of compounds generated by LC/TOF-MS. J Biomol Tech 19: 258–266. 19137116 PMC2567134

[8] Gardner M, Hall N, Fung E, White O, Berriman M, et al (2002) Genome sequence of the human malaria parasite Plasmodium falciparum. Nature 419: 498–511. 12368864 10.1038/nature01097 PMC3836256

[9] Hall N, Karras M, Raine J, Carlton J, Kooij T, et al (2005) A comprehensive survey of the Plasmodium life cycle by genomic, transcriptomic, and proteomic analyses. Science 307: 82–86. 15637271 10.1126/science.1103717

[10] Bozdech Z, Llinás M, Pulliam B, Wong E, Zhu J, et al (2003) The transcriptome of the intraerythrocytic developmental cycle of Plasmodium falciparum. PLoS Biol 1: E5. 12929205 10.1371/journal.pbio.0000005 PMC176545

[11] Foth B, Zhang N, Mok S, Preiser P, Bozdech Z (2008) Quantitative protein expression profiling reveals extensive post-transcriptional regulation and post-translational modifications in schizont-stage malaria parasites. Genome Biol 9: R177. 19091060 10.1186/gb-2008-9-12-r177 PMC2646281

[12] Ginsburg H (2006) Progress in in silico functional genomics: the malaria Metabolic Pathways database. Trends Parasitol 22: 238–240. 16707276 10.1016/j.pt.2006.04.008

[13] Kanehisa M, Goto S (2000) KEGG: kyoto encyclopedia of genes and genomes. Nucleic Acids Res 28: 27–30. 10592173 10.1093/nar/28.1.27 PMC102409

[14] Venter JC, Adams MD, Myers EW, Li PW, Mural RJ, et al (2001) The sequence of the human genome. Science 291: 1304–1351. 11181995 10.1126/science.1058040

[15] Aurrecoechea C, Brestelli J, Brunk BP, Dommer J, Fischer S, et al (2009) PlasmoDB: a functional genomic database for malaria parasites. Nucleic Acids Res 37: D539–543. 18957442 10.1093/nar/gkn814 PMC2686598

[16] Sana TR, Waddell K, Fischer SM (2008) A sample extraction and chromatographic strategy for increasing LC/MS detection coverage of the erythrocyte metabolome. J Chromatogr B Analyt Technol Biomed Life Sci 871: 314–321. 10.1016/j.jchromb.2008.04.030 18495560

[17] Pesek JJ, Matyska MT, Fischer SM, Sana TR (2008) Analysis of hydrophilic metabolites by high-performance liquid chromatography-mass spectrometry using a silica hydride-based stationary phase. J Chromatogr A 1204: 48–55. 18701108 10.1016/j.chroma.2008.07.077

[18] Pesek JJ, Matyska MT, Loo JA, Fischer SM, Sana TR (2009) Analysis of hydrophilic metabolites in physiological fluids by HPLC-MS using a silica hydride-based stationary phase. J Sep Sci 32: 2200–2208. 19569099 10.1002/jssc.200900270

[19] Technologies A (2008) Agilent Fiehn GC/MS Metabolomics RTL Library.

[20] Sana TR, Fischer S, Wohlgemuth G, Katrekar A, Jung KH, et al (2010) Metabolomic and transcriptomic analysis of the rice response to the bacterial blight pathogen Xanthomonas oryzae pv. oryzae. Metabolomics 6: 451–465. 20676379 10.1007/s11306-010-0218-7 PMC2899020

[21] Trager W, Jensen JB (1976) Human malaria parasites in continuous culture. Science 193: 673–675. 781840 10.1126/science.781840

[22] Palmer KL, Hui GS, Siddiqui WA, Palmer EL (1982) A large-scale in vitro production system for Plasmodium falciparum. J Parasitol 68: 1180–1183. 6757400

[23] Foth BJ, Zhang N, Chaal BK, Sze SK, Preiser PR, et al.. (2011) Quantitative time-course profiling of parasite and host cell proteins in the human malaria parasite Plasmodium falciparum. Mol Cell Proteomics. 10.1074/mcp.M110.006411 PMC3149090 21558492

[24] Jackson KE, Spielmann T, Hanssen E, Adisa A, Separovic F, et al (2007) Selective permeabilization of the host cell membrane of Plasmodium falciparum-infected red blood cells with streptolysin O and equinatoxin II. Biochem J 403: 167–175. 17155936 10.1042/BJ20061725 PMC1828889

[25] Canales J, Fernández A, Rodrigues J, Ferreira R, Ribeiro J, et al (2009) Hydrolysis of the phosphoanhydride linkage of cyclic ADP-ribose by the Mn(2+)-dependent ADP-ribose/CDP-alcohol pyrophosphatase. FEBS Lett 583: 1593–1598. 19379742 10.1016/j.febslet.2009.04.023

[26] Fiehn O, Kopka J, Trethewey RN, Willmitzer L (2000) Identification of uncommon plant metabolites based on calculation of elemental compositions using gas chromatography and quadrupole mass spectrometry. Anal Chem 72: 3573–3580. 10952545 10.1021/ac991142i

[27] Kind T, Wohlgemuth G, Lee dY, Lu Y, Palazoglu M, et al (2009) FiehnLib: mass spectral and retention index libraries for metabolomics based on quadrupole and time-of-flight gas chromatography/mass spectrometry. Anal Chem 81: 10038–10048. 19928838 10.1021/ac9019522 PMC2805091

[28] Hochberg Y, Benjamini Y (1990) More powerful procedures for multiple significance testing. Stat Med 9: 811–818. 2218183 10.1002/sim.4780090710

[29] Tukey JW (1949) Comparing individual means in the analysis of variance. Biometrics 5: 99–114. 18151955

[30] Kind T, Fiehn O (2006) Metabolomic database annotations via query of elemental compositions: mass accuracy is insufficient even at less than 1 ppm. BMC Bioinformatics 7: 234. 16646969 10.1186/1471-2105-7-234 PMC1464138

[31] van Iersel MP, Pico AR, Kelder T, Gao J, Ho I, et al (2010) The BridgeDb framework: standardized access to gene, protein and metabolite identifier mapping services. BMC Bioinformatics 11: 5. 20047655 10.1186/1471-2105-11-5 PMC2824678

[32] Teng R, Junankar PR, Bubb WA, Rae C, Mercier P, et al (2009) Metabolite profiling of the intraerythrocytic malaria parasite Plasmodium falciparum by (1)H NMR spectroscopy. NMR Biomed 22: 292–302. 19021153 10.1002/nbm.1323

[33] Olszewski KL, Morrisey JM, Wilinski D, Burns JM, Vaidya AB, et al (2009) Host-parasite interactions revealed by Plasmodium falciparum metabolomics. Cell Host Microbe 5: 191–199. 19218089 10.1016/j.chom.2009.01.004 PMC2737466

[34] Wishart DS, Knox C, Guo AC, Eisner R, Young N, et al (2009) HMDB: a knowledgebase for the human metabolome. Nucleic Acids Res 37: D603–610. 18953024 10.1093/nar/gkn810 PMC2686599

[35] Guse AH (2000) Cyclic ADP-ribose. J Mol Med 78: 26–35. 10759027 10.1007/s001090000076

[36] Jones ML, Cottingham C, Rayner JC (2009) Effects of calcium signaling on Plasmodium falciparum erythrocyte invasion and post-translational modification of gliding-associated protein 45 (PfGAP45). Mol Biochem Parasitol 168: 55–62. 19576251 10.1016/j.molbiopara.2009.06.007 PMC2754074

[37] Olszewski KL, Morrisey JM, Wilinski D, Burns JM, Vaidya AB, et al (2009) Host-parasite interactions revealed by Plasmodium falciparum metabolomics. Cell Host Microbe 5: 191–199. 19218089 10.1016/j.chom.2009.01.004 PMC2737466

[38] Sana T, Fischer S, Tichy S (2010) An LC/MS Metabolomics Discovery Workflow for Malaria-Infected Red Blood Cells Using Mass Profiler Professional Software and LC-Triple Quadrupole MRM Confirmation. Santa Clara, CA: Agilent Technologies, Inc.

[39] Olszewski KL, Mather MW, Morrisey JM, Garcia BA, Vaidya AB, et al (2010) Branched tricarboxylic acid metabolism in Plasmodium falciparum. Nature 466: 774–778. 20686576 10.1038/nature09301 PMC2917841

[40] Olszewski KL, Llinás M (2010) Central carbon metabolism of Plasmodium parasites. Mol Biochem Parasitol. 10.1016/j.molbiopara.2010.09.001 PMC3004993 20849882

[41] Roth E (1990) Plasmodium falciparum carbohydrate metabolism: a connection between host cell and parasite. Blood Cells 16: 453–460; discussion 461–456. 2257322

[42] Kanaani J, Ginsburg H (1991) Transport of lactate in Plasmodium falciparum-infected human erythrocytes. J Cell Physiol 149: 469–476. 1660483 10.1002/jcp.1041490316

[43] Huang da W, Sherman BT, Lempicki RA (2009) Systematic and integrative analysis of large gene lists using DAVID bioinformatics resources. Nat Protoc 4: 44–57. 19131956 10.1038/nprot.2008.211

[44] Friend SH, Dai H (2006) Accelerating drug discovery: Open source cancer cell biology? Cancer Cell 10: 349–351. 17097556 10.1016/j.ccr.2006.10.011

[45] Bowen BP, Fischer CR, Baran R, Banfield JF, Northen T (2011) Improved genome annotation through untargeted detection of pathway-specific metabolites. BMC Genomics 12 Suppl 1S6. 10.1186/1471-2164-12-S1-S6 PMC3223729 21810208

[46] Yeh I, Hanekamp T, Tsoka S, Karp PD, Altman RB (2004) Computational analysis of Plasmodium falciparum metabolism: organizing genomic information to facilitate drug discovery. Genome Res 14: 917–924. 15078855 10.1101/gr.2050304 PMC479120

[47] Roberts CW, Roberts F, Lyons RE, Kirisits MJ, Mui EJ, et al (2002) The shikimate pathway and its branches in apicomplexan parasites. J Infect Dis 185 Suppl 1S25–36. 11865437 10.1086/338004

[48] Elford BC, Haynes JD, Chulay JD, Wilson RJ (1985) Selective stage-specific changes in the permeability to small hydrophilic solutes of human erythrocytes infected with Plasmodium falciparum. Mol Biochem Parasitol 16: 43–60. 3897858 10.1016/0166-6851(85)90048-9

[49] Downie MJ, Kirk K, Mamoun CB (2008) Purine salvage pathways in the intraerythrocytic malaria parasite Plasmodium falciparum. Eukaryot Cell 7: 1231–1237. 18567789 10.1128/EC.00159-08 PMC2519781

[50] Gowda DC, Davidson EA (1999) Protein glycosylation in the malaria parasite. Parasitol Today 15: 147–152. 10322336 10.1016/s0169-4758(99)01412-x

[51] Takasawa S, Tohgo A, Noguchi N, Koguma T, Nata K, et al (1993) Synthesis and hydrolysis of cyclic ADP-ribose by human leukocyte antigen CD38 and inhibition of the hydrolysis by ATP. J Biol Chem 268: 26052–26054. 8253715

[52] Currie KP, Swann K, Galione A, Scott RH (1992) Activation of Ca(2+)-dependent currents in cultured rat dorsal root ganglion neurones by a sperm factor and cyclic ADP-ribose. Mol Biol Cell 3: 1415–1425. 1283541 10.1091/mbc.3.12.1415 PMC275709

[53] Bailey VC, Sethi JK, Fortt SM, Galione A, Potter BV (1997) 7-Deaza cyclic adenosine 5′-diphosphate ribose: first example of a Ca(2+)-mobilizing partial agonist related to cyclic adenosine 5′-diphosphate ribose. Chem Biol 4: 51–61. 9070427 10.1016/s1074-5521(97)90236-2

[54] Jones M, Cottingham C, Rayner J (2009) Effects of calcium signaling on Plasmodium falciparum erythrocyte invasion and post-translational modification of gliding-associated protein 45 (PfGAP45). Mol Biochem Parasitol 168: 55–62. 19576251 10.1016/j.molbiopara.2009.06.007 PMC2754074

[55] Galione A (1992) Ca(2+)-induced Ca2+ release and its modulation by cyclic ADP-ribose. Trends Pharmacol Sci 13: 304–306. 1413089 10.1016/0165-6147(92)90096-o

[56] Lakshmanan V, Rhee KY, Wang W, Yu Y, Khafizov K, et al (2012) Metabolomic analysis of patient plasma yields evidence of plant-like α-linolenic acid metabolism in Plasmodium falciparum. J Infect Dis 206: 238–248. 22566569 10.1093/infdis/jis339 PMC3490690

[57] de Carvalho LP, Zhao H, Dickinson CE, Arango NM, Lima CD, et al (2010) Activity-based metabolomic profiling of enzymatic function: identification of Rv1248c as a mycobacterial 2-hydroxy-3-oxoadipate synthase. Chem Biol 17: 323–332. 20416504 10.1016/j.chembiol.2010.03.009 PMC2878197

