# Supplementary figures and images for: Global Mass Spectrometry Based Metabolomics Profiling of Erythrocytes Infected with Plasmodium falciparum
Source: PLoS One. 2013 Apr 9;8(4):e60840. doi: 10.1371/journal.pone.0060840 (PMC3621881; doi:10.1371/journal.pone.0060840)

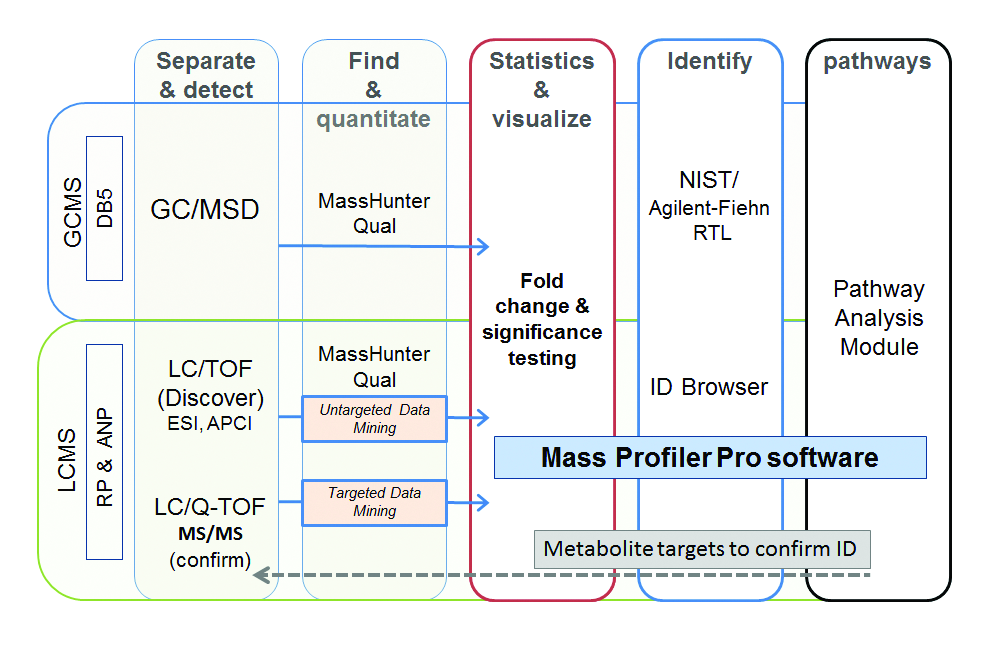

Supplement: Figure S1 — Untargeted profiling workflow for LC/MS and GC/MS separation, detection, processing, and annotation of data which can then be used for mapping onto metabolic pathways. (TIF) [file pone.0060840.s001.tif]

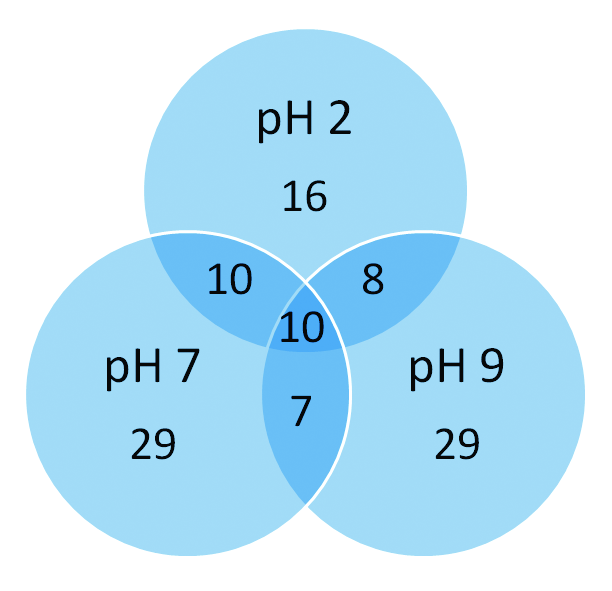

Supplement: Figure S2 — Venn diagram of the distribution of differential annotated features between NRBC and IRBC groups for pH 2, 7 and 9 extraction conditions ( P< 0.05). (TIF) [file pone.0060840.s002.tif]

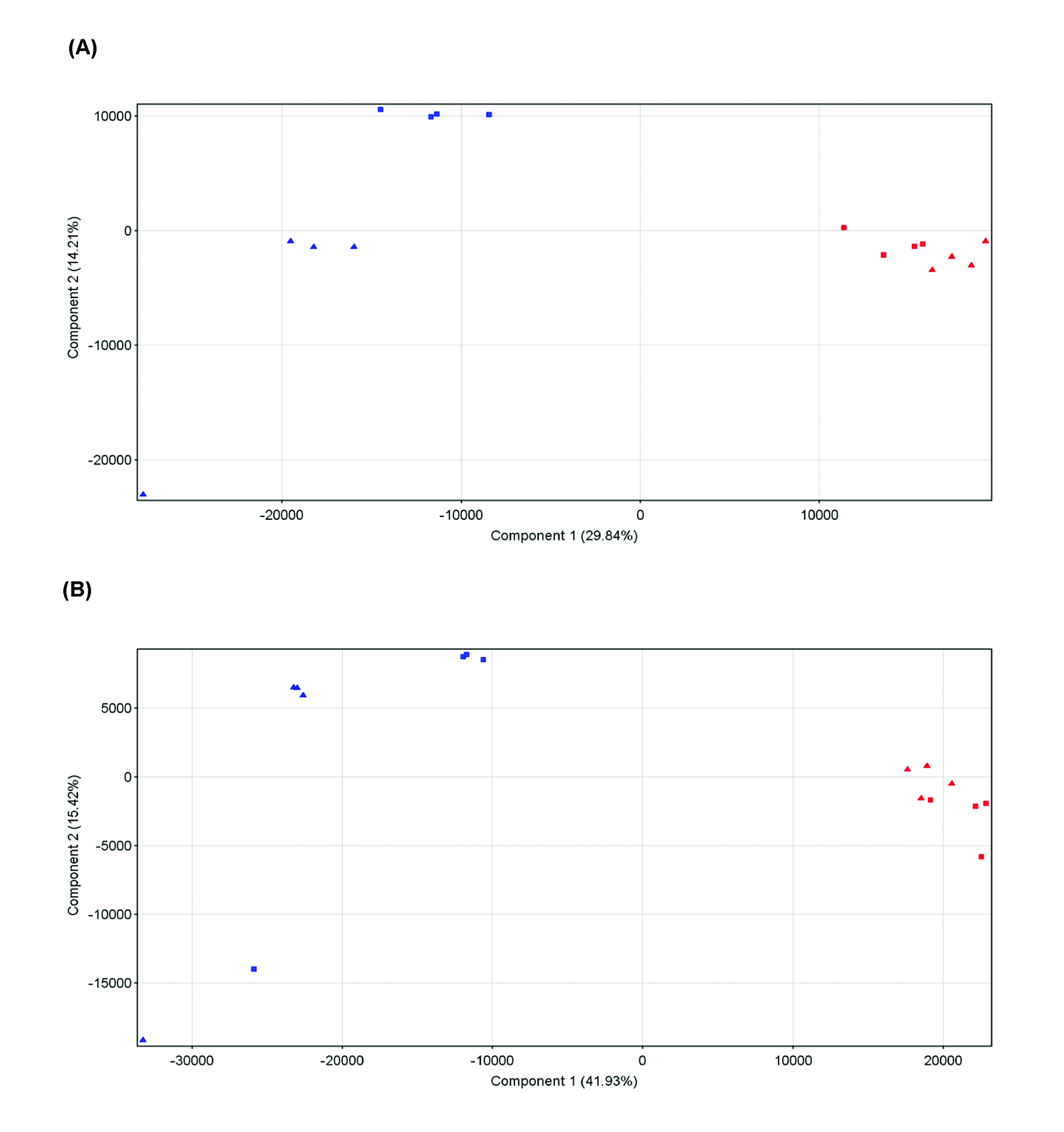

Supplement: Figure S3 — (A) 2D PCA plot of pH 7 solvent extracts for 662 metabolites analyzed in ESI positive ion mode and (B) 547 metabolites analyzed in ESI negative ion mode (See Table 1 ). IRBC: red filled squares, 0 SLO; red filled triangles 250 U SLO; NRBC: blue filled squares, 0 SLO; blue filled triangles 250 U SLO. (TIF) [file pone.0060840.s003.tif]

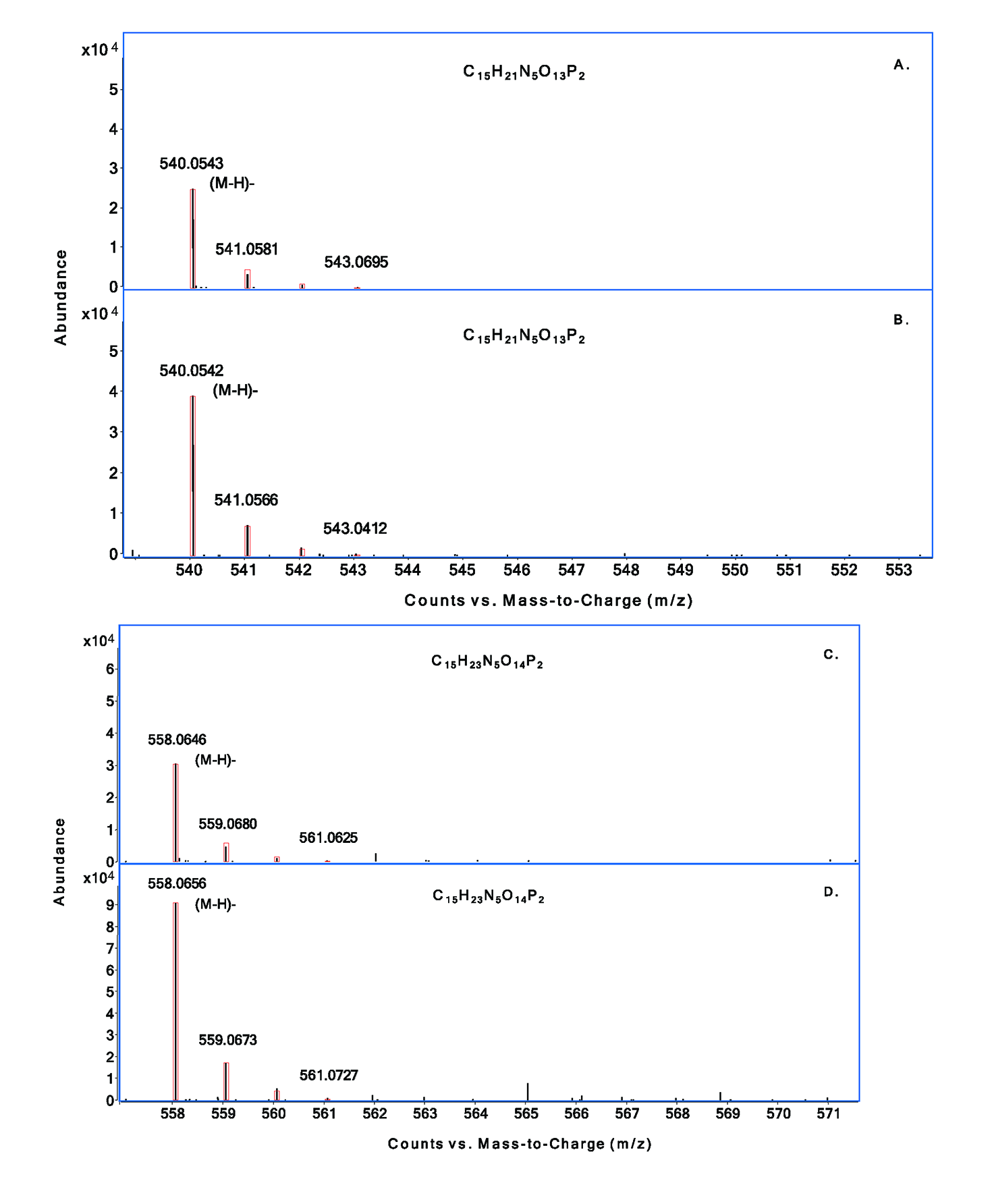

Supplement: Figure S4 — LC/MS ESI (−) extracted spectral scan comparisons for cADPR and pRib-AMP. (A) cADPR: an incomplete in vitro chemical derivitization reaction for cADPR or C15H21N5O13P2 (m/z 540.0536); (B) cADPR in a NRBC sample; and for (C) pRib-AMP or C15H23N5O14P2: the extracted spectrum for the derivitized product (m/z 558.0649); (D) extracted spectrum for pRib-AMP in an IRBC sample. Black lines are instrument measured isotopes. Height and spacing between the red boxes correspond to the theoretical, MFG calculated isotopes for each empirical formula. (TIF) [file pone.0060840.s004.tif]

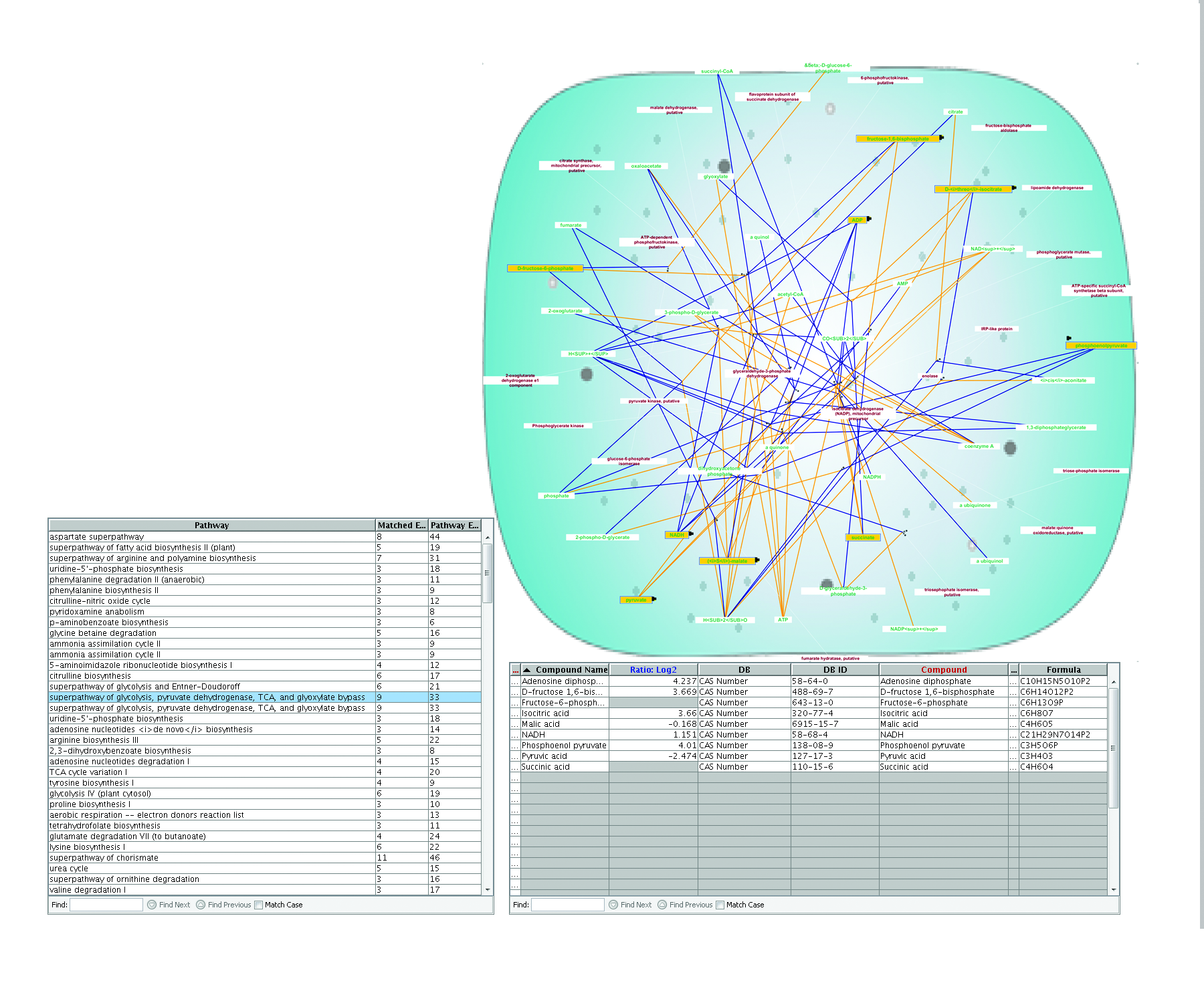

Supplement: Figure S5 — Pathway analysis of the annotated metabolites for P. falciparum infected RBC cultures was based on querying BioCyc pathways for P. falciparum 3D7 strain in Mass Profiler Professional software. The representative figure and tables are an example, depicting the pathway network and compound matches associated with the superpathway for glycolysis, pyruvate dehydrogenase, TCA and glyoxylate bypass. (TIF) [file pone.0060840.s005.tif]
