# Supplementary material for: Global Mass Spectrometry Based Metabolomics Profiling of Erythrocytes Infected with Plasmodium falciparum
Source: PLoS One. 2013 Apr 9;8(4):e60840. doi: 10.1371/journal.pone.0060840 (PMC3621881; doi:10.1371/journal.pone.0060840)
Supplement: Table S1 — Summary of results for differentially-expressed features based on Fold Change (FC) and t-test comparisons between NRBC and IRBC groups. (DOCX) [file pone.0060840.s006.docx]

**Table S1.** Summary of results for differentially-expressed features based on Fold Change (FC) and *t*-test comparisons between NRBC and IRBC groups.

|  | **pH2** | **pH7** | **pH9** | **pH2** | **pH7** | **pH9** |
| --- | --- | --- | --- | --- | --- | --- |
|  | *RP/ESI-* | *RP/ESI-* | *RP/ESI-* | *ANP/ESI-* | *ANP/ESI-* | *ANP/ESI-* |
| **FC > 2** | 317 | 324 | 235 | NA | 41 | 35 |
| **P < 0.05 Infection State** | 96 | 112 | 37 | NA | 34 | 33 |
| **Expected by Chance** | 7 | 7 | 1 | NA | 2 | 3 |
|  |  |  |  |  |  |  |
|  | *RP/ESI+* | *RP/ESI+* | *RP/ESI+* | *ANP/ESI+* | *ANP/ESI+* | *ANP/ESI+* |
|  | 370 | 336 | 391 | 54 | 52 | 31 |
|  | 89 | 115 | 62 | 27 | 19 | 7 |
|  | 4 | 7 | 3 | 3 | 1 | 0 |
|  |  |  |  |  |  |  |
|  | *APCI+* | *APCI+* | *APCI+* |  |  |  |
|  | 110 | 118 | 117 |  |  |  |
|  | 48 | 30 | 45 |  |  |  |
|  | 2 | 1 | 8 |  |  |  |

* The Input Feature lists for calculation of the *P*-value were from Table 1, where each feature had a CV < 100% across each sample group. Fold Change (FC) and *P* values were computed from Volcano plots, invoking an un-paired *t*-test with equal variance between sample groups and the Benjamini Hochberg (reference) FDR
