# Supplementary material for: Global Mass Spectrometry Based Metabolomics Profiling of Erythrocytes Infected with Plasmodium falciparum
Source: PLoS One. 2013 Apr 9;8(4):e60840. doi: 10.1371/journal.pone.0060840 (PMC3621881; doi:10.1371/journal.pone.0060840)
Supplement: Table S2 — Untargeted and targeted mining results were combined and used for differential analysis of metabolite data acquired by global untargeted analysis using RP/LC-ESI and APCI. (DOCX) [file pone.0060840.s007.docx]

**Table S2.** Untargeted and targeted mining results were combined and used for differential analysis of metabolite data acquired by global untargeted analysis using RP/LC-ESI and APCI.

Heat map key for Fold Change:

| **No.** | **Formula** | **Mass** | **METLIN matches*** | **Compound^#^** | **METLIN** | **CAS or HMDB** | **[IRBC/NRBC]**  **Log2** |
| --- | --- | --- | --- | --- | --- | --- | --- |
| 1 | C6H6N2O | 122.0480 | 1 | Niacinamide | 1497 | 98-92-0 | **2.26** |
| 2 | C5H5N5 | 135.0548 | 1 | Adenine | 85 | 73-24-5 | **1.50** |
| 3 | C6H14N4O2 | 174.1118 | 1 | Arginine | 13 | 74-79-3 | **-17.76** |
| 4 | C3H7O7P | 185.9926 | 1 | D-Glycerate 3-phosphate | 150 | 3443-57-0 | **18.53** |
| 5 | C11H23NO2 | 201.1724 | 1 | 11-amino-undecanoic acid | 35923 | - | **-0.02** |
| 6 | C12H25NO2 | 215.1877 | 1 | 12-amino-dodecanoic acid | 35924 | - | **-0.36** |
| 7 | C11H20O4 | 216.1360 | 1 | Undecanedioic acid | 5846 | HMDB00888 | **-0.02** |
| 8 | C9H17NO5 | 219.1107 | 1 | Pantothenic Acid | 241 | 137-08-6 | **16.27** |
| 9 | C9H13N3O4 | 227.0901 | 1 | Deoxycytidine | 3367 | 951-77-9 | **0.74** |
| 10 | C13H24O4 | 244.1670 | 1 | 2-methyl-dodecanedioic acid | 35968 | HMDB02327 | **0.39** |
| 11 | C10H18NO6 | 248.1134 | 1 | Malonylcarnitine** | 6484 | - | **-0.33** |
| 12 | C12H22O5 | 246.1458 | 1 | 3-Hydroxydodecanedioic acid | 5402 | HMDB00413 | **-0.14** |
| 13 | C8H16N2O6S | 268.0729 | 1 | Homolanthionine | 6449 | HMDB02034 | **-1.95** |
| 14 | C10H17N3O6S | 307.0842 | 1 | Glutathione | 44 | 70-18-8 | **0.49** |
| 15 | C11H17N3O7S | 335.0797 | 1 | S-Formylglutathione | 3469 | 50409-81-9 | **-0.26** |
| 16 | C10H13N4O8P | 348.0486 | 1 | Inosine 5'-monophosphate (IMP) | 3490 | 131-99-7 | **-16.69** |
| 17 | C9H15N4O9P | 354.0565 | 1 | 5-Amino-6-(5'-phosphoribosylamino)uracil | 3497 | - | **-0.99** |
| 18 | C18H38NO5P | 379.2504 | 1 | Sphingosine-1-phosphate | 3891 | 26993-30-6 | **-14.13** |
| 19 | C26H50O4 | 426.3708 | 1 | Hexacosanedioic acid | 35992 | - | **-0.22** |
| 20 | C17H22N4O8S | 442.1167 | 1 | S-(4-Nitrobenzyl)glutathione | 4098 | 6803-19-6 | **0.08** |
| 21 | C24H49O9P | 512.3103 | 1 | GPGro(18:0/0:0)[U] | 40877 | - | **14.38** |
| 22 | C15H21N5O13P2 | 541.0607 | 1 | Cyclic adenosine diphosphate ribose | 4171 | 119340-53-3 | **13.84** |
| 23 | C21H29N7O14P2 | 665.1244 | 1 | NADH | 3687 | 58-68-4 | **2.22** |
| 24 | C21H28N7O17P3 | 743.0758 | 1 | NADP+ | 102 | 53-57-6 | **0.74** |
| 25 | C39H74O11P2 | 780.4701 | 1 | GPP(18:1(9Z)/18:1(9Z)) | 40951 | - | **-0.44** |
| 26 | C5H9NO2 | 115.0633 | 2 | Proline | 29 | 147-85-3 | **1.40** |
| 27 | C6H5NO2 | 123.0324 | 2 | Niacin (Nicotinic acid) | 240 | 59-67-6 | **0.85** |
| 28 | C5H12N2O2 | 132.0896 | 2 | Ornithine | 27 | 70-26-8 | **0.87** |
| 29 | C5H4N4O | 136.0382 | 2 | Hypoxanthine | 83 | 68-94-0 | **-0.88** |
| 30 | C11H9NO2 | 187.0630 | 2 | Indoleacrylic acid | 5702 | 1204-06-4 | **-0.58** |
| 31 | C15H12O | 208.0887 | 2 | Chalcone | 3342 | 94-41-7 | **-0.50** |
| 32 | C3H8O10P2 | 265.9591 | 2 | 1,3-Diphospho-D-Glyceric Acid | 153 | 138-81-8 | **18.69** |
| 33 | C11H14N4O4 | 266.1020 | 2 | 1-(3-Carboxypropyl)-3,7-dimethylxanthine | 1770 | 6493-07-8 | **19.27** |
| 34 | C7H15O10P | 290.0404 | 2 | Sedoheptulose 7-phosphate | 3418 | 2646-35-7 | **15.94** |
| 35 | C18H36O3 | 300.2663 | 2 | 11-hydroxy stearic acid | 35441 | HMDB10737 | **0.01** |
| 36 | C19H36O3 | 312.2667 | 2 | 18-oxo-nonadecanoic acid | 35819 | - | **-0.25** |
| 37 | C18H36O4 | 316.2610 | 2 | 7,8-dihydroxy stearic acid | 35465 | - | **-0.26** |
| 38 | C19H38O4 | 330.2767 | 2 | 1-hexadecanoyl-sn-glycerol | 75555 | HMDB11564 | **0.09** |
| 39 | C21H40O3 | 340.2979 | 2 | 20-oxo-heneicosanoic acid | 35826 | - | **-0.20** |
| 40 | C20H40O4 | 344.2923 | 2 | 1-heptadecanoyl-rac-glycerol | 4248 | 68002-72-2 | **-15.79** |
| 41 | C21H42O4 | 358.3082 | 2 | 1-octadecanoyl-rac-glycerol | 4249 | HMDB17575 | **-0.05** |
| 42 | C24H48O2 | 368.3650 | 2 | 22-methyl-tricosanoic acid | 4300 | - | **-0.12** |
| 43 | C22H42O4 | 370.3094 | 2 | Docosanedioic acid | 35989 | - | **-0.50** |
| 44 | C21H47NO6P | 440.3141 | 2 | GPCho(O-12:0/O-1:0) | 40186** | - | **0.65** |
| 45 | C15H23N5O14P2 | 559.0709 | 2 | N1-(5-Phospho-D-ribosyl)-AMP | 3596 | 1109-75-7 | **16.02** |
| 46 | C15H24N2O17P2 | 566.0545 | 2 | UDP-glucose | 3598 | 133-89-1 | **1.06** |
| 47 | C6H11NO2 | 129.0788 | 3 | Pipecolic acid | 50 | 535-75-1 | **0.66** |
| 48 | C4H9N3O2 | 131.0695 | 3 | Creatine | 7 | 6020-87-7 | **-1.39** |
| 49 | C3H5O6P | 167.9821 | 3 | Phosphoenol pyruvate | 152 | 138-08-9 | **19.27** |
| 50 | C8H12O4 | 172.0737 | 3 | 2-Octenedioic acid | 5330 | 5698-50-0 | **0.11** |
| 51 | C8H14O4 | 174.0888 | 3 | 2-Propylglutaric acid | 2998 | 32806-62-5 | **-0.04** |
| 52 | C8H16O4 | 176.1047 | 3 | 6,8-dihydroxy-octanoic acid | 35591 | - | **0.04** |
| 53 | C11H8O3 | 188.0474 | 3 | 1-Hydroxy-2-naphthoic acid | 2389 | 86-48-6 | **2.16** |
| 54 | C10H20O3 | 188.1406 | 3 | 3-Hydroxycapric acid | 6544 | - | **1.19** |
| 55 | C10H18O4 | 202.1206 | 3 | Sebacic acid | 4240 | 111-20-6 | **0.30** |
| 56 | C13H20O3 | 224.1411 | 3 | Methyl jasmonate | 3361 | 39924-52-2 | **0.28** |
| 57 | C12H22O4 | 230.1513 | 3 | Dodecanedioic acid | 5596 | HMDB00623 | **0.22** |
| 58 | C6H15O9P | 262.0453 | 3 | Mannitol 1-phosphate | 3393 | 15806-48-1 | **16.07** |
| 59 | C17H24O6 | 324.1575 | 3 | Idebenone Metabolite (QS-8) | 759 | 84166-50-7 | **0.24** |
| 60 | C24H46O4 | 398.3393 | 3 | Tetracosanedioic acid | 35991 | - | **0.00** |
| 61 | C5H10O2 | 102.0680 | 4 | Isovaleric acid | 109 | 503-74-2 | **-0.06** |
| 62 | C12H24O2 | 200.1773 | 4 | Lauric acid | 357 | 143-07-7 | **0.20** |
| 63 | C15H33NO7P | 370.2013 | 4 | GPCho(7:0/0:0[U]) | 40331** | - | **-0.17** |
| 64 | C16H35NO7P | 384.2150 | 4 | GPCho(8:0/0:0) | 40332** | - | **0.13** |
| 65 | C6H12O2 | 116.0832 | 5 | Hexanoic Acid | 111 | 142-62-1 | **0.05** |
| 66 | C9H11NO3 | 181.0739 | 5 | Tyrosine | 34 | 60-18-4 | **0.34** |
| 67 | C11H12N2O2 | 204.0896 | 5 | Tryptophan | 33 | 73-22-3 | **-0.51** |
| 68 | C13H24O3 | 228.1723 | 5 | 10-keto tridecanoic acid | 35736 | - | **0.28** |
| 69 | C14H28O2 | 228.2078 | 5 | 4,8-dimethyl-dodecanoic acid | 4315 | - | **-0.06** |
| 70 | C18H34O2 | 282.2559 | 5 | 5-octadecylenic acid (oleic acid) | 34752 | HMDB00207 | **-0.45** |
| 71 | C18H36O2 | 284.2722 | 5 | Stearic acid | 189 | 57-11-4 | **-0.40** |
| 72 | C19H38O2 | 298.2867 | 5 | 11-methyl-octadecanoic acid | 34637 | - | **-0.48** |
| 73 | C20H40O2 | 312.3026 | 5 | Isoarachidic acid | 4296 | 6250-72-2 | **-0.51** |
| 74 | C10H15N5O10P2 | 427.0292 | 5 | Adenosine diphosphate | 34522 | 58-64-0 | **18.86** |
| 75 | C6H13NO2 | 131.0945 | 6 | Leucine | 24 | 61-90-5 | **-0.47** |
| 76 | C9H8O3 | 164.0474 | 6 | Coumaric acid | 307 | 501-98-4 | **-0.25** |
| 77 | C10H20O2 | 172.1461 | 6 | 3-methyl-nonanoic acid | 4489 | - | **0.07** |
| 78 | C10H18O3 | 186.1254 | 6 | 2-oxo capric acid | 35701 | - | **0.09** |
| 79 | C11H22O2 | 186.1619 | 6 | Undecanoic acid | 4203 | 112-37-8 | **-0.42** |
| 80 | C15H30O2 | 242.2243 | 6 | Pentadecylic acid | 4205 | 98-79-3 | **-0.05** |
| 81 | C10H14N5O7P | 347.0622 | 6 | Adenosine monophosphate | 34478 | 149022-20-8 | **0.06** |
| 82 | C7H7NO2 | 137.0475 | 7 | 2-Pyridylacetic acid | 1308 | 13115-43-0 | **0.34** |
| 83 | C7H10O4 | 158.0579 | 7 | 4,6-dioxoheptanoic acid | 4154 | 51568-18-4 | **0.31** |
| 84 | C9H18O2 | 158.1304 | 7 | (+)-6-methyl caprylic acid | 4225 | - | **0.22** |
| 85 | C16H25N5O15P2 | 589.0827 | 7 | Guanosine diphosphofucose | 4187 | HMDB01095 | **15.21** |
| 86 | C5H11NO2 | 117.0790 | 8 | Valine | 35 | 72-18-4 | **0.03** |
| 87 | C9H8O2 | 148.0529 | 8 | Cinnamic acid | 310 | 621-82-9 | **0.03** |
| 88 | C6H10O3 | 130.0628 | 9 | 2-oxoisocaproic acid | 121 | 816-66-0 | **0.31** |
| 89 | C6H10O4 | 146.0579 | 9 | Adipic acid | 115 | 124-04-9 | **-0.29** |
| 90 | C12H20O3 | 212.1410 | 9 | 12-oxo-10Z-dodecenoic acid | 74732 | - | **0.24** |
| 91 | C18H32O2 | 280.2395 | 9 | 13E,17-octadecadienoic acid | 34967 | - | **-0.70** |
| 92 | C9H11NO2 | 165.0786 | 10 | Phenylalanine | 28 | 63-91-2 | **-0.57** |
| 93 | C20H26O3 | 314.1880 | 10 | 4-Ketoretinoic acid | 2282 | 38030-57-8 | **0.21** |
| 94 | C6H12O5 | 164.0683 | 11 | Beta-D-Fucose | 3284 | 28161-52-6 | **0.35** |
| 95 | C6H13O9P | 260.0310 | 11 | myo-inositol 3-phosphate | 58553 | 2831-74-5 | **15.03** |
| 96 | C8H12O2 | 140.0834 | 13 | 2-octynoic acid | 35231 | - | **0.82** |
| 97 | C20H32O6 | 368.2197 | 13 | PGG2 | 3508 | 51982-36-6 | **-0.15** |
| 98 | C9H10O2 | 150.0678 | 14 | Hydrocinnamic acid | 4153 | 501-52-0 | **-0.03** |
| 99 | C10H18O2 | 170.1302 | 14 | 9-Decenoic acid | 34521 | 14436-32-9 | **0.09** |
| 100 | C12H20O2 | 196.1461 | 14 | 4-dodecynoic acid | 35121 | - | **0.09** |
| 101 | C16H32O2 | 256.2411 | 21 | Palmitic acid | 187 | 57-10-3 | **-0.39** |
| 102 | C20H32O5 | 352.2246 | 24 | Thromboxane A2 | 6253 | HMDB01452 | **0.38** |
| 103 | C20H30O2 | 302.2249 | 33 | Timnodonic acid (EPA) | 6423 | 10417-94-4 | **0.23** |
| 104 | C15H26O | 222.1993 | 45 | 2,4-pentadecadienal | 36605 | HMDB30938 | **0.33** |

The results include the empirical formula, the number of unique METLIN database matches (< 5 ppm tolerance), and log_2_ transformed “Fold Change” ratios between IRBC/NRBC groups.

* Number of matches based on 2012 METLIN database and corrected for duplicates, stereoisomers, and synthetic compounds.

^#^ Compounds with more than one annotation per formula, either the first one on the METLIN list is displayed, or the annotation with most compelling biological relevance was selected for representation. These annotations were used to investigate subsequent pathway enrichment.

** Cation
