# Supplementary material for: Global Mass Spectrometry Based Metabolomics Profiling of Erythrocytes Infected with Plasmodium falciparum
Source: PLoS One. 2013 Apr 9;8(4):e60840. doi: 10.1371/journal.pone.0060840 (PMC3621881; doi:10.1371/journal.pone.0060840)
Supplement: Table S3 — Differential analysis for metabolite data acquired by global untargeted analysis using ANP/LC-ESI. The results for untargeted (MFE) and targeted (FbF) data mining strategies are shown. (DOCX) [file pone.0060840.s008.docx]

**Table S3.** Differential analysis for metabolite data acquired by global untargeted analysis using ANP/LC-ESI. The results for untargeted (MFE) and targeted (FbF) data mining strategies are shown.

Heat map key for Fold Change:

**MFE**

| **No.** | **Formula** | **Mass** | **METLIN db matches*** | | **Compound#** | **METLIN** | **CAS or**  **HMDB** | | **[IRBC/NRBC] Log2** |
| --- | --- | --- | --- | --- | --- | --- | --- | --- | --- |
| 1 | C2H7NO3S | 125.0147 | 1 | | Taurine | 31 | 107-35-7 | | **-14.70** |
| 2 | C9H17NO2 | 171.1259 | 1 | | Gabapentin | 2989 | 60142-96-3 | | **0.61** |
| 3 | C6H14N4O2 | 174.1117 | 1 | | Arginine | 13 | 74-79-3 | | **-3.95** |
| 4 | C16H33NO | 255.2562 | 1 | | Palmitic amide | 62905 | 629-54-9 | | **0.46** |
| 5 | C13H21N3O8S | 379.1049 | 1 | | S-Lactoylglutathione | 3511 | 41656-56-8 | | **-0.03** |
| 6 | C18H38NO5P | 379.2488 | 1 | | Sphingosine 1-phosphate | 3891 | 26993-30-6 | | **-0.12** |
| 7 | C23H43NO5 | 413.3141 | 1 | | 3-Hydroxy-9-hexadecenoylcarnitine* | 75472 | HMDB 13333 | | **0.63** |
| 8 | C5H12N2O2 | 132.0899 | 2 | | Ornithine | 27 | 70-26-8 | | **1.15** |
| 9 | C10H13N5O | 219.1120 | 2 | | Cis-zeatin | 64074 | HMDB 12204 | | **-0.93** |
| 10 | C8H16N2O4S2 | 268.0552 | 2 | | Homocystine | 4189 | 462-10-2 | | **13.57** |
| 11 | C6H13N3O3 | 175.0957 | 2 | | Citrulline | 16 | 372-75-8 | | **12.61** |
| 12 | C5H11NO2S | 149.0511 | 3 | | Methionine | 26 | 63-68-3 | | **18.63** |
| 13 | C6H9N3O2 | 155.0695 | 3 | | Histidine | 21 | 71-00-1 | | **16.13** |
| 14 | C3H5O6P | 167.9824 | 3 | | Phosphoenol pyruvate | 152 | 138-08-9 | | **16.11** |
| 15 | C7H8N4O2 | 180.0647 | 3 | | Paraxanthine | 1456 | 83-67-0 | | **-0.54** |
| 16 | C18H20N2O6 | 360.1321 | 3 | | Dityrosine | 58352 | 980-21-2 | | **0.34** |
| 17 | C4H7NO4 | 133.0375 | 4 | | Aspartic acid | 15 | 56-84-8 | | **-0.26** |
| 18 | C3H7O6P | 169.9980 | 4 | | Glyceraldehyde 3-phosphate | 3294 | 591-57-1 | | **14.12** |
| 19 | C21H42O4 | 358.3083 | 4 | | MG(18:0/0:0/0:0) | 4249 | HMDB 11131 | | **-0.51** |
| 20 | C34H52O8 | 588.3662 | 4 | | 25-Hydroxyvitamin D2-25-glucuronide | 61661 | HMDB 10342 | | **0.80** |
| 21 | C7H6O2 | 122.0368 | 5 | | Benzoic acid | 1297 | 65-85-0 | | **0.54** |
| 22 | C4H8O5 | 136.0372 | 5 | | Threonic acid | 35473 | HMDB 00943 | | **1.18** |
| 23 | C19H38O4 | 330.2770 | 5 | | MG(0:0/16:0/0:0) | 62317 | HMDB 11533 | | **-0.61** |
| 24 | C19H40NO7P | 425.2542 | 5 | | LysoPE(0:0/14:0) | 62258 | HMDB 11470 | | **0.60** |
| 25 | C8H12O4 | 172.0736 | 6 | | 2-Octenedioic acid | 5330 | 5698-50-0 | | **0.02** |
| 26 | C4H6O5 | 134.0215 | 7 | | Malic acid | 118 | 6915-15-7 | | **0.89** |
| 27 | C6H14N2O2 | 146.1055 | 7 | | Lysine | 25 | 56-87-1 | | **0.75** |
| 28 | C9H11NO3 | 181.0739 | 7 | | Tyrosine | 34 | 60-18-4 | | **0.63** |
| 29 | C11H12N2O2 | 204.0899 | 7 | | Tryptophan | 33 | 73-22-3 | | **0.41** |
| 30 | C5H11NO2 | 117.0790 | 8 | | Valine | 35 | 72-18-4 | | **-0.09** |
| 31 | C5H7NO3 | 129.0426 | 8 | | Pyroglutamic acid | 3251 | 98-79-3 | | **-0.24** |
| 32 | C4H8N2O3 | 132.0535 | 8 | | Asparagine | 14 | 70-47-3 | | **14.86** |
| 33 | C5H10N2O3 | 146.0691 | 8 | | Glutamine | 58182 | 53622-85-8 | | **-1.76** |
| 34 | C10H14N5O7P | 347.0631 | 8 | | Adenosine 5'-monophosphate | 34478 | 149022-20-8 | | **-0.49** |
| 35 | C21H38O4 | 354.2770 | 8 | | MG(0:0/18:2(9Z,12Z)/0:0) | 62322 | HMDB 11538 | | **-0.44** |
| 36 | C11H15NO2 | 193.1103 | 10 | | (S)-N-Methylsalsolinol | 58182 | 53622-85-8 | | **-0.15** |
| 37 | C6H10O7 | 194.0427 | 10 | | Glucuronic acid | 161 | 6556-12-3 | | **12.66** |
| 38 | C5H9NO4 | 147.0532 | 11 | | L-Glutamate | 19 | 56-86-0 | | **4.01** |
| 39 | C6H8O7 | 192.0270 | 11 | | Isocitric acid | 3328 | 320-77-4 | | **12.64** |
| 40 | C9H11NO2 | 165.0790 | 12 | | Phenylalanine | 65707 | 165.079 | | **0.27** |
| 41 | C6H13NO2 | 131.0946 | 13 | | Leucine | 23 | 73-32-5 | | **0.79** |
| 42 | C6H8O4 | 144.0423 | 13 | | 3-Methylglutaconic acid | 45911 | HMDB 00522 | | **-0.39** |
| 43 | C6H14O12P2 | 339.9961 | 13 | | D-fructose 1,6-bisphosphate | 147 | 488-69-7 | | **12.72** |
| 44 | C15H30O2 | 242.2246 | 15 | | Pentadecanoic acid | 4205 | 1002-84-2 | | **0.06** |
| 45 | C5H8O5 | 148.0372 | 17 | | 2-Hydroxyglutaric acid | 45120 | 103404-90-6 | | **4.25** |
| 46 | C6H10O6 | 178.0477 | 17 | | 2-Keto-3-deoxy-D-gluconic acid | 6184 | HMDB 01353 | | **1.49** |
| 47 | C14H28O2 | 228.2089 | 26 | | Myristic acid | 196 | 544-63-8 | | **0.12** |
| 48 | C20H28O2 | 300.2089 | 32 | | 9-cis-Retinoic acid | 41515 | 5300-03-8 | | **0.09** |
| 49 | C16H32O2 | 256.2402 | 35 | | Palmitic acid | 187 | 57-10-3 | | **-1.07** |
| 50 | C27H44O4 | 432.3239 | 58 | | 24-Hydroxycalcitriol | 58368 | 56142-94-0 | | **-0.11** |
| 51 | C25H35O4 | 399.25354 | NA | | 11'-carboxy-gama-tocotrienol | - | HMDB 12518 | | **-0.31** |
| 52 | C20H31O5 | 351.21716 | NA | | 13,14-Dihydro-15-oxo-lipoxin A4 | - | HMDB 12564 | | **-0.59** |
| **FbF** | |  | |  |  |  |  | |  |
| **No.** | **Formula** | **Mass** | **METLIN db matches*** | | **Compound^#^** | **METLIN** | **CAS or**  **HMDB** | **[IRBC/NRBC] Log2** | |
| 1 | C5H5N5 | 135.0545 | | 1 | Adenine | 85 | 73-24-5 | | **0.32** |
| 2 | C6H14N4O2 | 174.1117 | | 1 | Arginine | 13 | 74-79-3 | | **-3.33** |
| 3 | C9H17NO5 | 219.1107 | | 1 | Pantothenate | 241 | 137-08-6 | | **0.82** |
| 4 | C5H12O8P2 | 262.0007 | | 1 | 1-Hydroxy-2-methyl-2-butenyl4-diphosphate | 53272 | - | | **0.67** |
| 5 | C11H20N2O6 | 276.1321 | | 1 | N6-(L-1,3-Dicarboxypropyl)-L-lysine | 63454 | 997-68-2 | | **19.32** |
| 6 | C10H17N3O6S | 307.0838 | | 1 | Glutathione | 44 | 70-18-8 | | **0.58** |
| 7 | C14H14N6O3 | 314.1127 | | 1 | Dihydropteroate | 3444 | 2134-76-1 | | **0.17** |
| 8 | C10H13N4O8P | 348.0471 | | 1 | IMP | 3490 | 131-99-7 | | **-1.70** |
| 9 | C19H23N7O6 | 445.1710 | | 1 | Tetrahydrofolate | 714 | 135-16-0 | | **-1.05** |
| 10 | C33H34N4O6 | 582.2478 | | 1 | Biliverdin IX | 6608 | 114-25-0 | | **0.23** |
| 11 | C20H32N6O12S2 | 612.1520 | | 1 | Glutathionedisulfide | 45 | 27025-41-8 | | **-0.20** |
| 12 | C32H60NO11P | 665.3904 | | 1 | 1-Palmitoyl-2-(5-hydroxy-8-oxo-6-octenedioyl)-sn-glycero-3-phosphatidylcholine | 69737 | - | | **0.08** |
| 13 | CH4N2O | 60.0324 | | 2 | Urea | 6 | 57-13-6 | | **-0.08** |
| 14 | C5H9N | 83.0735 | | 2 | Piperideine | 63458 | - | | **0.51** |
| 15 | C3H7NO3 | 105.0426 | | 2 | Serine | 30 | 56-45-1 | | **-0.49** |
| 16 | C5H9NO2 | 115.0633 | | 2 | Proline | 29 | 147-85-3 | | **-0.70** |
| 17 | C5H12N2O2 | 132.0899 | | 2 | Ornithine | 27 | 70-26-8 | | **0.68** |
| 18 | C4H7NO4 | 133.0375 | | 2 | Aspartate | 63417 | 1783-96-6 | | **-0.33** |
| 19 | C5H4N4O | 136.0385 | | 2 | Hypoxanthine | 83 | 68-94-0 | | **-0.39** |
| 20 | C5H11NO2S | 149.0510 | | 2 | Methionine | 26 | 63-68-3 | | **0.24** |
| 21 | C3H10NO4P | 155.0347 | | 2 | N-Methylethanolaminephosphate | 63347 | - | | **0.05** |
| 22 | C3H9O6P | 172.0137 | | 2 | Glycerol 3-phosphate | 5161 | HMDB 00126 | | **18.72** |
| 23 | C10H11NO5 | 225.0637 | | 2 | 4-Amino-4-deoxychorismate | 3363 | 133442-18-9 | | **0.09** |
| 24 | C10H10O6 | 226.0477 | | 3 | Chorismate | 62850 | 55508-12-8 | | **-0.45** |
| 25 | C10H12N4O5 | 268.0808 | | 3 | Inosine | 84 | 58-63-9 | | **-17.45** |
| 26 | C34H40N4O4 | 568.3050 | | 3 | Protoporphyrinogen IX | 6003 | HMDB 01097 | | **0.15** |
| 27 | C2H5NO2 | 75.0320 | | 4 | Glycine | 20 | 56-40-6 | | **0.07** |
| 28 | C3H7O6P | 169.9980 | | 4 | Glyceraldehyde3-phosphate | 149 | 57-04-5 | | **0.76** |
| 29 | C8H13NO4 | 187.0845 | | 4 | 6-Acetamido-2-oxohexanoate | 66023 | HMDB 12150 | | **-0.13** |
| 30 | C5H6O5 | 146.0215 | | 5 | 2-Oxoglutarate | 119 | 328-50-7 | | **1.12** |
| 31 | C6H14N2O2 | 146.1055 | | 5 | Lysine | 25 | 56-87-1 | | **0.55** |
| 32 | C9H11NO3 | 181.0739 | | 5 | Tyrosine | 34 | 60-18-4 | | **-0.24** |
| 33 | C33H36N4O6 | 584.2635 | | 5 | Bilirubin | 81 | 635-65-4 | | **0.03** |
| 34 | C6H13NO2 | 131.0946 | | 6 | Leucine | 24 | 61-90-5 | | **0.13** |
| 35 | C8H9NO4 | 183.0532 | | 6 | 4-Pyridoxate | 239 | 82-82-6 | | **-14.74** |
| 36 | C10H14N5O7P | 347.0631 | | 6 | Adenosine monophosphate | 34478 | 149022-20-8 | | **1.63** |
| 37 | C5H10N2O3 | 146.0691 | | 7 | Glutamine | 18 | 56-85-9 | | **-1.57** |
| 38 | C5H11NO2 | 117.0790 | | 8 | Valine | 35 | 72-18-4 | | **0.06** |
| 39 | C4H8N2O3 | 132.0535 | | 8 | Asparagine | 14 | 70-47-3 | | **0.00** |
| 40 | C5H9NO4 | 147.0532 | | 9 | Glutamate | 19 | 56-86-0 | | **7.82** |
| 41 | C9H11NO2 | 165.0790 | | 9 | Phenylalanine | 28 | 63-91-2 | | **-0.16** |
| 42 | C8H9NO3 | 167.0582 | | 9 | Pyridoxal | 2203 | 66-72-8 | | **-0.73** |
| 43 | C7H7NO2 | 137.0477 | | 10 | 4-Aminobenzoate | 3261 | 150-13-0 | | **-0.09** |
| 44 | C7H13NO3 | 159.0895 | | 13 | 5-Acetamidopentanoate | 63464 | HMDB 12175 | | **-0.27** |
| 45 | C6H14O12P2 | 339.9961 | | 13 | 1D-myo-inositol1,4-bisphosphate | 410 | HMDB 00968 | | **-0.36** |
| 46 | C5H9NO3 | 131.0582 | | 16 | 4-Hydroxy-L-proline | 58354 | 30724-02-8 | | **-0.06** |
| 47 | C6H12O6 | 180.0634 | | 24 | myo-Inositol | 144 | 87-9-8 | | **-0.44** |
| 48 | C6H13O9P | 260.0297 | | 36 | Fructose 6-phosphate | 146 | 643-13-0 | | **-0.44** |

The results include the empirical formula, the number of unique METLIN database matches (<5 ppm tolerance), and log_2 -_transformed “Fold Change” ratios between IRBC/NRBC groups.

* Number of matches based on 2012 METLIN database and corrected for duplicates, stereoisomers, and synthetic compounds.

^#^ Compounds with more than one annotation per formula, either the first one on the METLIN list is displayed, or the annotation with most compelling biological significance was selected for representation. These annotations were used to investigate subsequent pathway enrichment.
