# Supplementary material for: Global Mass Spectrometry Based Metabolomics Profiling of Erythrocytes Infected with Plasmodium falciparum
Source: PLoS One. 2013 Apr 9;8(4):e60840. doi: 10.1371/journal.pone.0060840 (PMC3621881; doi:10.1371/journal.pone.0060840)
Supplement: Table S4 — List of metabolites confirmed by MS/MS spectral matching and/or retention time (RT) matching to chemical standards. (DOCX) [file pone.0060840.s009.docx]

**Table S4.** List of metabolites confirmed by MS/MS spectral matching and/or retention time (RT) matching to chemical standards.

| **Compound** | **Mass** | **Formula** | **MS/MS and/or RT match confirmation** |
| --- | --- | --- | --- |
| Proline | 115.06333 | C5H9NO2 | MS/MS |
| Niacinamide | 122.04801 | C6H6N2O | MS/MS |
| Pyroglutamic acid | 129.04259 | C5H7NO3 | MS/MS |
| Leucine | 131.09463 | C6H13NO2 | MS/MS |
| Ornithine | 132.08988 | C5H12N2O2 | MS/MS |
| Hypoxanthine | 136.03851 | C5H4N4O | MS/MS |
| Arginine | 174.11168 | C6H14N4O2 | MS/MS |
| Citrulline | 175.0957 | C6H13N3O3 | MS/MS |
| Palmitic acid | 256.24023 | C16H32O2 | RT |
| Adenosine monophosphate | 347.06308 | C10H14N5O7P | RT |
| cyclic adenosine diphosphate ribose | 541.06111 | C15H21N5O13P2 | MS/MS |
| NADPH | 743.07545 | C21H28N7O17P3 | MS/MS |
| N1-(5-Phospho-D-ribosyl)-AMP | 559.07167 | C15H23N5O14P2 | MS/MS |
