# Supplementary material for: Global Mass Spectrometry Based Metabolomics Profiling of Erythrocytes Infected with Plasmodium falciparum
Source: PLoS One. 2013 Apr 9;8(4):e60840. doi: 10.1371/journal.pone.0060840 (PMC3621881; doi:10.1371/journal.pone.0060840)
Supplement: Table S5 — List of metabolites confirmed by Agilent Fiehn GC/MS Metabolomics RTL Library. (DOCX) [file pone.0060840.s010.docx]

**Table S5.** List of metabolites confirmed by Agilent Fiehn GC/MS Metabolomics RTL Library.

| **Compound** | **RT (min.)** | **Score (DB)** | **Formula** | **Log_2_ [IRBC/NRBC]*** |
| --- | --- | --- | --- | --- |
| 2-hydroxybutyric acid | 8.06 | 56.06 | C4H8O3 | -0.16 |
| Benzoic acid | 9.59 | 72.18 | C7H6O2 | **IRBC** |
| D-(+) galactose | 17.39 | 78.54 | C6H12O6 | **IRBC** |
| D-allose | 17.39 | 92.62 | C6H12O6 | **-1.00** |
| D-glucose | 17.38 | 92.39 | C6H12O6 | **-1.81** |
| DL-glyceraldehyde | 9.14 | 61.05 | C3H6O3 | 0.40 |
| D-threitol | 13.00 | 69.22 | C4H10O4 | -0.65 |
| Fructose | 17.12 | 51.63 | C6H12O6 | 0.24 |
| Glycolic acid | 7.11 | 92.69 | C2H4O3 | 0.08 |
| L-(+) lactic acid | 6.88 | 72.69 | C3H6O3 | -0.12 |
| L-tyrosine | 17.87 | 55.9 | C9H11NO3 | **IRBC** |
| Palmitic acid | 18.88 | 88.54 | C16H32O2 | 0.16 |
| Porphine | 10.74 | 75.62 | C20H14N4 | 0.20 |
| Pyruvic acid | 6.82 | 62.82 | C3H4O3 | 0.18 |
| Stearic acid | 20.70 | 87.9 | C18H36O2 | -0.08 |
| Succinic acid | 10.56 | 51.33 | C4H6O4 | -0.13 |
| Tagatose | 17.18 | 53.06 | C6H12O6 | **NRBC** |
| Talose | 17.59 | 84.22 | C6H12O6 | **-1.07** |

*Compounds with IRBC or NRBC notation were detected only in those extracts.
