# Supplementary material for: Global Mass Spectrometry Based Metabolomics Profiling of Erythrocytes Infected with Plasmodium falciparum
Source: PLoS One. 2013 Apr 9;8(4):e60840. doi: 10.1371/journal.pone.0060840 (PMC3621881; doi:10.1371/journal.pone.0060840)
Supplement: Methods S1 — Supplementary Materials and Methods. (DOCX) [file pone.0060840.s011.docx]

**Materials and Methods Supplement**

**RP LC/MS**

The resuspended aqueous phase sample extracts were analyzed by RP in ESI +, ESI- and APCI+. Although we analyzed the different solvent pH extracts separately, we recommend combining the trio of pH 2, 7 and 9 sample extracts corresponding to each condition. This dramatically reduces the number of samples to analyze, and more importantly, aids in the interpretation of results. RP LC separation was performed with an Agilent 1200 LC (Agilent Technologies, Santa Clara, CA, USA). RP separation was achieved by injecting 1 μL on a ZORBAX SB-Aq column (2.1 x 50 mm, 1.8 μm), preceded in series with a ZORBAX -SB-C8 Rapid Resolution Cartridge (2.1 x 30mm, 3.5 um). The Agilent 6520 Quadropole Time-of-Flight LC/MS (Q-TOF) was equipped with an electrospray (ESI) ion source or an atmospheric pressure chemical ionization (APCI) ion source (*see Supplement_material-methods for further details*). The organic phase extracts containing a mixture of chloroform and methanol, were centrifuged at 16,000 g, 4 °C for 10 min. in order to remove any cellular debris. The supernatants were dried briefly in a SpeedVac, resuspended in LC solvent and analyzed by APCI, primarily for the analysis of neutral molecules, such as lipids, as it causes little or no fragmentation of the analyte, and is suitable for volatile and thermally stable compounds.

**ANP LC/MS**

Examples of classes that can be separated in complex biological matrixes using the ANP technique are sugars (neutral), amino acids (basic) and organic acids (acidic). The retention mechanism of acidic and basic compounds on the silica hydride column is highly pH dependent, whereas neutrals are not. Therefore, two separate, general purpose ANP chromatographic methods were developed to achieve retention and separation of (1) neutral and basic compounds by (+) ESI-MS, and (2) neutral and acidic compounds by (-) ESI-MS on a Cogent Diamond Hydride (150mm×2.1mm) column (MicroSolv Technologies, Eatontown, NJ).. High acetonitrile to water gradients were used for both methods. Method (1) used a buffered ammonium acetate mobile phase, at neutral pH; with a pH gradient generated by the addition of increasing formic acid. Bottle A: 50:50 methanol/ water and 0.05% formic acid; Bottle B: 90% acetonitrile / 10% water 10 mM NH_4_AcO. Gradient (min., %B): (0, 100%), (12, 50%), (14, 50%), (15, 0%), (19, 0%), (20, 100%). Flow Rate: 0.4 ml/min, Stop time: 20 min, Post time: 5 min. Method (2) used a simple pH system with a constant amount of formic acid. Bottle A: 50:50 methanol/ water and 0.1% formic acid Bottle B: 100% acetonitrile with 0.10% formic acid. Gradient: (0, 95%), (1, 95%), (15, 20%), Flow Rate: 0.6 ml/min, Stop time: 15 min, Post time: 5 min. Since we observed that ammonia permanently alters the physio-chemical properties of the silica hydride surface, a separate column was dedicated for each method. We used HDPE bottles to minimize the presence of sodium ions leached from glass bottles, resulting in dramatic improvements in peak shape. (*see Supplement_material-methods for further details*)

**GC/MS Data Acquisition and Peak Finding**

The organic phases from each of the individual metabolite extracts were dried and derivatized in two steps according to (Fiehn et al. Anal. Chem. 2000, 72, 3573-3580) : Methoxyamination to protect the carbonyl groups, followed by trimethysilylation to decrease the boiling point. Thus methoxyamine hydrochloride in pyridine, and then using N-methyl-N-(1) trimethylsilyltrifluoroacetamide containing 1% trimethylchlorosilane. (2) Myristic-d27 acid was used as internal standard. For untargeted GC/MS metabolite separation, we equipped an Agilent 7890 GC with a 30 m long Agilent DB-5MS column with 10 m Duragard pre-column; with 0.25 µm film thickness and 250 µm diameter was used for the separation. All GC-MS experiments were performed as described in the Agilent user manual (Agilent G1676AA Fiehn GC/MS metabolomics RTL library user guide). Four biological replicates were analyzed for each sample and the data was extracted using both AMDIS (NIST) spectral deconvolution software, as well as Agilent “Find by Chromatographic Deconvolution” software.

**cADPR and pRib-AMP**

Cyclic adenosine diphosphate ribose (cADPR) and all other chemical standards were purchased from Sigma-Aldrich (St. Louis, MO). Standard stock solutions of 1 mg/ml in methanol were prepared and diluted for analysis. N1(5-phosphoribosyl)-AMP (pRib-AMP) was prepared from a cADPR standard by hydrolysis of the cADPR phosphoanhydride linkage. The cADPR standard and the derivatized product containing a mixture of cADPR and pRib-AMP, were each diluted 100-fold prior to injection onto an Agilent 1200 LC/QTOF system and detected by ESI (-) mode. These two compounds eluted at different retention times from the column. MS/MS spectra were collected at 10, 20 and 40 eV collision energies. Their MS/MS spectra were added to a library of over 2700 MS/MS spectra, that were edited and annotated using Agilent PCDL Manager software. Spectral matching was accomplished by searching the corresponding peaks in the library and unknown spectra with a default tolerance for precursor ion set at symmetric +/-10 ppm, and for the product ion it was set at symmetric +/-20 ppm. When a corresponding peak was found in the library, a dot product of the library peak intensity and unknown peak intensity was calculated. A matching score was generated by summing the dot products for all the peaks in a given spectra, and normalized to produce a score between 0 and 100.

**LC/MS Data Acquisition Parameters**

The LC parameters were: autosampler temperature, 4 °C; injection volume, 1 μL; column temperature, 60 °C; and flow rate of 0.6 mL/min. The LC solvent and timeframe was a 2% to 98% linear gradient of water with 0.2% acetic acid (A) and methanol with 0.2% acetic acid (B), employed over 13 minutes followed by a solvent hold for 19 minutes, at which time data collection was stopped. A 5 minute post-time for column re-equilibration was included.

For the LC-QTOF, the nitrogen was used as the instrument gas. For ESI, source voltage (Vcap) was 4,000 V in positive mode and 3,500 V in negative ion mode. The fragmentor was set to 140 V for both ion polarities. The drying gas temperature was 250 °C; drying gas flow was 10 l/min; and nebulizer pressure was 45 psi. The ESI source used a separate nebulizer for the continuous, low-level (10 μL/min) introduction of reference mass compounds.

For APCI analysis, the same LC solvent conditions as ESI were used. APCI capillary voltage was 4000V, corona current was 4μA, and the fragmentor was set to 170V. The nebulizer was 60 psig and the nitrogen drying gas was set to a flow rate of 5 L/min. Drying gas temperature and vaporizer temperature were maintained at 250 °C and 350 °C, respectively. The reference compounds were introduced by merging the effluent of the HPLC with a low flow introduction of reference mass compounds.

The reference mass ions used were 121.050873, 922.009798 (positive ion mode) and 119.036320, 966.000725 (negative ion mode). The reference mass compounds are used to maintain mass axis calibration. Data was collected at an acquisition rate of 2 Hz in both positive and negative ion modes from m/z 50 to 1000. Data was stored in both profile and centroid modes.

**Feature Finding**

Definitions vary widely in the literature for what a chemical entity represents at different stages of a workflow. In this manuscript, we use the term “feature” to describe chemical entities that are found as a result of using an unbiased (untargeted) peak finding algorithm. We refer to compounds or provisionally identified metabolites when we have an empirical formula assigned to the feature by an “annotated” database match. These compounds have an accurate mass within a specified mass tolerance window, a corresponding empirical formula calculated from the abundance and distribution of isotopes that matched one or more annotations in the METLIN database. These compounds possess an accurate mass within a specified mass tolerance window, and a corresponding empirical formula, that was matched to a single annotation in the METLIN database We use the term “metabolite” when unequivocal evidence for its identity has been confirmed, by accurate mass and retention time matching to a database of standards (AMRT), and/or by MS/MS spectral library matching. “Untargeted” data acquisition on the LC/Q-TOF was performed using Agilent MassHunter™ (“Acquisition”) B.03.01 software (Agilent Technologies, Santa Clara, CA, USA). The data was deconvoluted into individual chemical peaks in Agilent MassHunter™ Qualitative Analysis B.03.01 (“MassHunter™ Qual”), using Molecular Feature Extractor (MFE), a naïve or “untargeted” data-mining algorithm.

A separate, complementary “targeted” data mining approach was used that was based on a list of annotated compounds with empirical formulas. An algorithm, Find by Molecular Formula was used to find compounds in LC/MS data files that are specifically known to be associated with *P.* *falciparum*. The list of literature derived metabolite formulas was imported into a Personal Compound Database (PCD) and used for targeted data mining. The list of compounds and their associated formulas was derived from the publicly available, MPMP database [1]. The elemental formulas for each compound in the target list were used to calculate the theoretical accurate mass for each entry and added to a customized, malaria specific, Personal Compound Database and Library (PCDL). The malaria PCDL was uploaded into MassHunter™ Qual, and was searched for PCDL matches to corresponding masses in the acquired data. The results of the analyses was a series of extracted ion chromatograms (EICs) that were saved as *xml* formatted files and were used for subsequent statistical analysis and data visualization in Mass Profiler Professional (MPP) software

**Untargeted Data Acquisition and Analysis**

The untargeted Molecular Feature Extraction (MFE) algorithm removes the background ions, locates the co-variant ions in a chromatogram, tests for chemically logical relationships, looks for user specified adducts (H+, Na+ and K+) and. groups together isotopes to form a single compound (feature) with unique neutral mass and retention time (RT) value. Charge state for metabolites was set to 1. Each processed data file containing the monoisotopic mass, total abundance and retention time information was saved as an *xml* formatted file for subsequent data anlaysis and visualization in Mass Profiler Professional (MPP).

**Untargeted Data Acquisition with “Targeted” Data Mining**

A separate, “targeted” discovery data mining approach was performed in MassHunter Qualtitative Analysis software using the Formula for the known compound to calculate an accurate mass so that an algorithm could search for the mass in the data. This generated extracted ion chromatograms (EICs) based on the list of target compounds which were derived from the MPMP database [1]. The list of elemental formulas for these compounds was used to build a custom, malaria specific, personal compound database (PCDL), and loaded into Agilent MassHunter™ Qual (B.03.01). Sample data files were queried against this database to find matches and *xml* based result files were generated for each sample for subsequent statistical analysis in Mass Profiler Professional software (MPP).

**Data Filtering, Statistical analysis and data visualization**

Separate projects were created in MPP based on the ionization mode and ion polarity of acquired data. The data were filtered to create entity (feature) lists based on the number of entities detected in at least one condition for the NRBC and IRBC sample replicates. For a given condition (i.e. pH), filtered entity lists were created based on the number of entities that passed the 100% or 75% sample replicate thresholds.

Four independent biological replicate samples were analyzed as one group after the ion intensity for each molecular ion was averaged across the replicates. The entities were first filtered for the frequency of detection in all biological replicates. The effectiveness of SLO treatment was determined by PCA analysis for all conditions at each pH. Differentially expressed feature lists between infected and non-infected cultures, with and without SLO treatment, were determined by ANOVA and identified using the METLIN PCDL.

The data files were aligned, and grouping of data files was according to condition, filtering, quality control, statistical analysis and compound identification via METLIN database matching. Unsupervised principal component analysis (PCA) was performed with mean centering and scaling to visually demonstrate the variance of the metabolic phenotypes within the group on the sample files based on the following variables: infection state, pH and SLO treatment. ANOVA analysis resulted in lists of differentially expressed features. They were provisionally identified by matching compounds to the METLIN database. Multivariate statistical analysis using unsupervised PCA was performed with mean centering and scaling to visually demonstrate the variance of the metabolic phenotypes from the groups based on the discriminating features from the ANOVA analysis

Statistical evaluation of the data was performed using univariate analyses, including the Welch’s unpaired t-test for independent pairs of groups, and one way ANOVA for multiple groups. The effects of infection state and extraction solvent pH on classifying the samples were compared. A cutoff value of *p <0.05* was considered statistically significant in one-way ANOVA, using the Benjamini and Hochberg False Discovery Rate was set to 5% for multiple testing corrections [2]. The difference between each pair of means with appropriate adjustment for multiple testing in the ANOVA was investigated with the Tukey multiple comparison test [3]. The results were summarized as a matrix table showing the comparison results for each pair of conditions as a *p-value*. Parameters were selected that computed the Fold Change and *p-*values

.

Missing values were separately included and then excluded from the calculations, and the results were compared. The former approach was relevant where a compound was present in “relative” amounts in both NRBC and IRBC samples; and the latter was used for “absolute” situations where an entity was present in one condition but not the other.

**Identification of annotated features**

To demonstrate the utility of an untargeted metabolomics analysis, provisional compound IDs were generated based on accurate mass, isotope ratios, abundance s and spacing, as well as RT matching for select compounds. The compounds found to be significantly different between IRBC and NRBC were identified in MPP by accurate mass matching, and/or with additional RT matching to a METLINdatabase of > 25,000 compounds . The Agilent METLIN database L currently contains over 25,000 compounds, and includes links to KEGG identifiers, CAS numbers, HMDB and LIPID MAPS identifiers.

.

**Provisional metabolite Annotation**

An independent best fit empirical formula calculation (Molecular Formula Generator tool or MFG) was performed for each feature using the mass spectral metadata: isotopic mass and isotope ratio information. Each proposed empirical formula was compared to the database match formula to verify the database match. Isomers and isobaric compounds, however, remained unresolved.

**Publicly accessible databases**

MPMP was used to compile a non-redundant list of 281 well annotated compounds with IUPAC names. The corresponding empirical formulas for these compounds were imported into a PCDL database. In addition, many compounds listed in the Malaria literature (but absent from MPMP) were added to the Malaria PCDL totaling 350 compounds. The Malaria PCDL was subsequently used as the source database for provisionally identifying compounds that were processed using the Find by Formula algorithm in MassHunter™ Qual.

**LC/MS/MS Analysis for ID confirmation**

The previously described chromatographic methods were used in tandem with a Q-TOF mass spectrometer, operated in positive and negative modes, at collision energies of 10, 20, and 40 eV. The quadrupole filter was set to transmit a peak width of 1 amu. Mass range was set to detect 50-1600 amu for MS only data, and 25-1600 amu for MS/MS data.

**Pathways**

Pathway Commons

**Publicly accessible databases**

We gratefully acknowledge usage of the following websites and databases for their publicly accessible information:

| Database | Website |
| --- | --- |
| WikiPathways [4] | <http://www.wikipathways.org/index.php/WikiPathways> |
| MetaCyc [5] | <http://metacyc.org/> |
| Kyoto Encyclopedia of Genes and Genomes (KEGG) [6] | <http://www.kegg.com/> |
| The Human Metabolome Database (HMDB), v 2.5 [7] | <http://hmdb.ca/> |
| Nature Lipidomics Gateway (LIPID MAPS) [8] | [www.lipidmaps.org](http://www.lipidmaps.org) |
| Malaria Parasite Metabolic Pathways (MPMP)* [1] | http://priweb.cc.huji.ac.il/malaria/ |

**LC/MS/MS Analysis for ID confirmation**

An LC-QTOF (6520) was used for confirmation of metabolite identity for differentially abundant compounds. We built a “preferred ion list” of compounds that were found to be differential or of biological interest and required confirmation. An inclusion list of m/z and RT values was created in MPP. Several NRBC and IRBC samples were re-analyzed on the targeted MS/MS analysis. MS/MS spectra were acquired at three collision energies (10, 20 and 40 eV) and the sample spectra were compared and matched to a library of standard spectra inMETLIN PCDL, generated from MS/MS spectra of approximately 2,700 chemical standards.

**Chemical Standards**

Cyclic adenosine diphosphate ribose (cADPR) and all other chemical standards were purchased from Sigma-Aldrich (St. Louis, MO). Standard stock solutions of 1 mg/ml in methanol were prepared and diluted for analysis. N1(5-phosphoribosyl)-AMP (p was synthesized from a cADPR standard by hydrolysis of the cADPR phosphoanhydride linkage [9]

1. Ginsburg H (2006) Progress in in silico functional genomics: the malaria Metabolic Pathways database. Trends Parasitol 22: 238-240.

2. Hochberg Y, Benjamini Y (1990) More powerful procedures for multiple significance testing. Stat Med 9: 811-818.

3. TUKEY JW (1949) Comparing individual means in the analysis of variance. Biometrics 5: 99-114.

4. Kelder T, van Iersel MP, Hanspers K, Kutmon M, Conklin BR, et al. (2012) WikiPathways: building research communities on biological pathways. Nucleic Acids Res 40: D1301-1307.

5. Caspi R, Altman T, Dreher K, Fulcher CA, Subhraveti P, et al. (2012) The MetaCyc database of metabolic pathways and enzymes and the BioCyc collection of pathway/genome databases. Nucleic Acids Res 40: D742-753.

6. Kanehisa M, Goto S (2000) KEGG: kyoto encyclopedia of genes and genomes. Nucleic Acids Res 28: 27-30.

7. Wishart DS, Knox C, Guo AC, Eisner R, Young N, et al. (2009) HMDB: a knowledgebase for the human metabolome. Nucleic Acids Res 37: D603-610.

8. Fahy E, Sud M, Cotter D, Subramaniam S (2007) LIPID MAPS online tools for lipid research. Nucleic Acids Res 35: W606-612.

9. Canales J, Fernández A, Rodrigues J, Ferreira R, Ribeiro J, et al. (2009) Hydrolysis of the phosphoanhydride linkage of cyclic ADP-ribose by the Mn(2+)-dependent ADP-ribose/CDP-alcohol pyrophosphatase. FEBS Lett 583: 1593-1598.
